# Discovery of DEBIC to correlate P-selectin inhibition and DNA intercalation in cancer therapy and complicated thrombosis

**DOI:** 10.18632/oncotarget.23151

**Published:** 2017-12-08

**Authors:** Haiyan Chen, Wenjing Wang, Xiaoyi Zhang, Shan Liu, Yaonan Wang, Haimei Zhu, Jianhui Wu, Yuji Wang, Ming Zhao, Shiqi Peng

**Affiliations:** ^1^ Beijing Area Major Laboratory of Peptide and Small Molecular Drugs, Engineering Research Center of Endogenous Prophylactic of Ministry of Education of China, Beijing Laboratory of Biomedical Materials, College of Pharmaceutical Sciences, of Capital Medical University, Beijing, China; ^2^ Department of Biomedical Science and Environmental Biology, Kaohsiung Medical University, Kaohsiung, Taiwan

**Keywords:** dimethyl bisindolediacetate, anti-tumor, anti-thrombosis, P-selectin, d(CGATCG)_2_

## Abstract

Arterial thrombosis is one of the major complications of cancer and can seriously worsen the prognosis of the patients. These clinical findings encouraged this paper to correlate P-selectin inhibition and DNA intercalation in cancer therapy and complicated thrombosis. By designing and docking 12 derivatives of bisindole- 2-carboxylic acids into the active sites of P-selectin and d(CGATCG)_2_ 9 derivatives were assigned to receive *in vivo* anti-tumor assay, and finally provided dimethyl 2,2'-[(2,2'-(ethane-1,1-diyl)bis(1*H*-indole-3,2-diyl)]diacetate (DEBIC) to receive assays. DEBIC intercalated DNA and inhibited proliferation of tumor cells but not non-tumor cells. It slowed tumor growth of S180 mice at a dose of 0.36 μmol/kg, and slowed tumor growth of A549 bearing BABL/C mice at a dose of 8.9 μmol/kg. DEBIC was also found to inhibit arterial thrombosis by down regulating P-selectin effectively at a dose of 0.36 μmol/kg.

## INTRODUCTION

Among recent developments in medicinal chemistry of alkaloids, both indole and bisindole are notable for their diversification in therapeutic application. While the conjugates of indole and chalcone are anti-inflammatory and antioxidant agents [1], and the conjugates of indole and pyridine are anti-tuberculosis agents [2], anti-tumor indoles attract major research interests. The monoterpene indoles are apoptosis inducers [3], the substituted indole-2-carboxylic acids and indole incorporated thiazolylcoumarins are capable of cleaving the DNA [4–5]. In addition to inhibiting tuberculosis infection [6], the derivatives of indole-2-carboxilic acids are myeloid cell leukemia-1 inhibitors [7], the bioisosteric trifluoromethyl and pentafluorosulfanyl indoles are the AAA ATPase p97 inhibitors [8], and indeno [1,2-*b*]indole-9,10-dione derivatives are the inhibitors of casein kinase II of human [9]. Bisindoles appear been uniquely suited for the development of anti-tumor candidates [10–16], but so far no study focuses on the derivatives of bisindole-2-carboxylic acids and thereby to discover their derivatives that has dual actions of slowing tumor growth and inhibiting complicated thrombosis. Arterial thrombosis is one of the major complications of cancer and can seriously worsen the prognosis of the patients. Both P-selectin and DNA are likely involved in the onset of cancer and the complication of arterial thrombosis. These clinical findings led to a hypothesis that by inhibiting P-selectin and intercalating DNA an agent could simultaneously slow the tumor growth and block the arterial thrombosis of cancer patients. In this context, this paper reported the docking based design of 12 bisindole-2-carboxylic acid derivatives, the anti-tumor assay of 9 derivatives *in vitro* and *in vivo*. The findings of dimethyl 2,2′- [(2,2′- (ethane-1,1-diyl)bis(1*H*-indole-3,2-diyl)]diacetate (DEBIC) from the *in vitro* assays of proliferation of the tumor and non-tumor cells, from the *in vitro* assays of P-selectin inhibition and DNA intercalation, from the *in vivo* assays of the tumor growth, from the *in vitro* assay of platelet activation and from the *in vivo* assay of the arterial thrombosis were also mentioned.

## RESULTS

### Design of bisindole-2-carboxilic acid derivatives as P-selectin inhibitors and DNA intercalators

**Figure 1:**
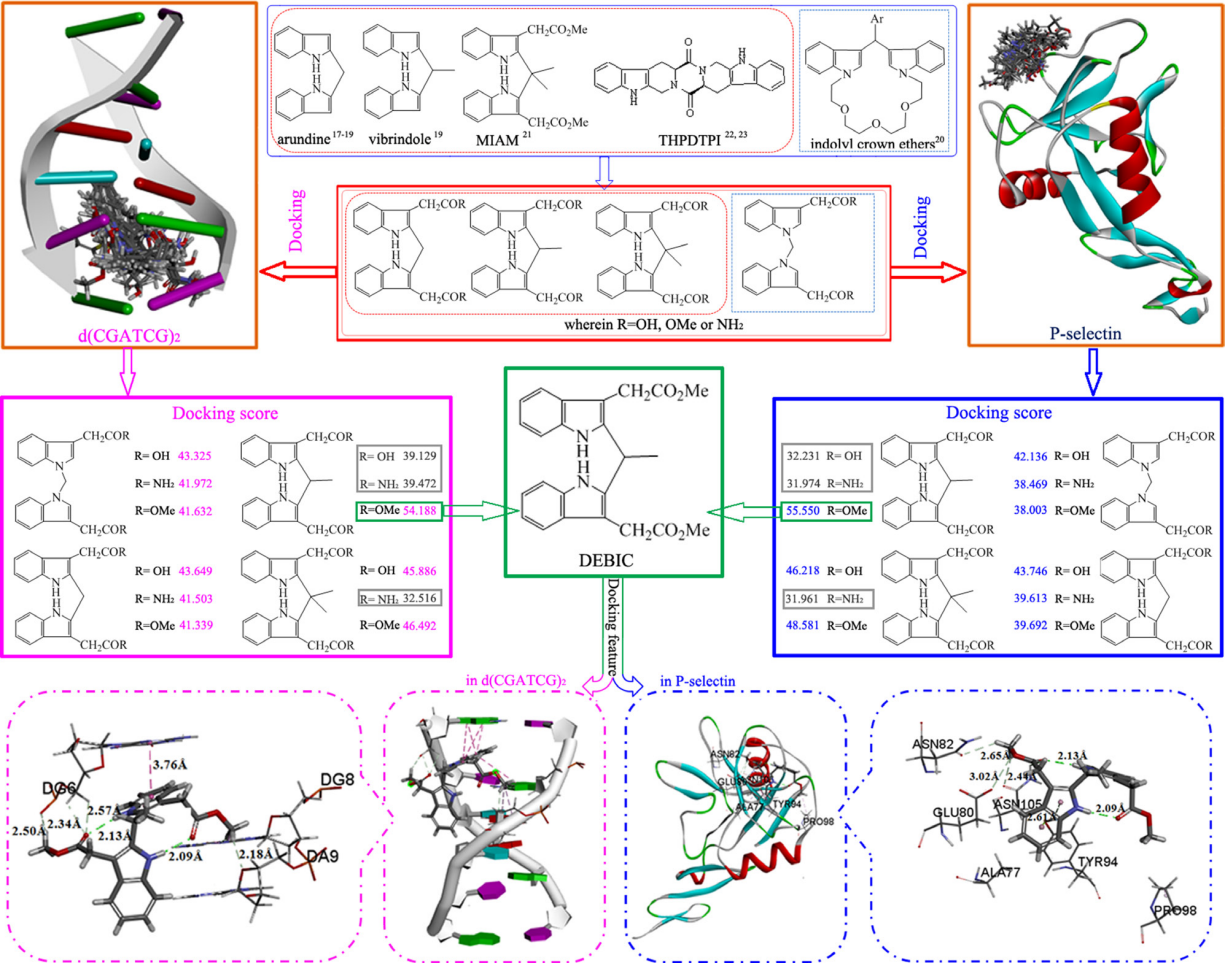
Design course: structural analysis of anti-tumor active indoles/bisindoles and DEBIC capable of targeting P-selectin and intercalating DNA provides 12 derivatives of bisindole-2- carboxylic acids, docking of them towards P-selectin and d(CGATCG)_2_

Structural analysis of anti-tumor active indoles [16–18], bisindoles [19–21], and THPDTPI capable of targeting P-selectin and DNA [22–23] led to a hypothesis that the derivatives of 2,2’-(1,1’-methylenebis(1*H*-indole-3,1-diyl))diacetic acid, 2,2’- [(2,2’- methylene)bis(1*H*-indole-3,2-diyl)]diacetic acid, 2,2’- [(2,2’-(ethane-1,1-diyl)bis(1*H*-indole-3,2-diyl)]diacetic acid and 2,2’-[(2,2’-(propane-2,2-diyl)bis(1*H*-indole-3,2-diyl)]diacetic acid could target P-selectin and intercalate DNA, thereby could slow tumor growth and inhibit thrombosis. This hypothesis was firstly examined by the docking of the derivatives towards the active sites of P-selectin and d(CGATCG)_2_ (Figure 1). The docking was performed with standard procedures [22–23] and the docking scores of the derivatives with P-selectin and d(CGATCG)_2_ were 32.0–55.6 and 32.5–54.2, respectively. Of them three derivatives (in gray box), the docking scores in P-selectin been less than 38 and the docking scores in d(CGATCG)_2_ been less than 41, were considered inactive and were not synthesized. On the other hand dimethyl 2,2’-[(2,2’-(ethane-1,1-diyl)bis(1*H*-indole-3,2-diyl)]diacetate (DEBIC), in green box, the docking score in P-selectin been 55.550 and the docking score been 54.188 in d(CGATCG)_2_, was considered the most active derivative having anti-tumor and anti-thrombotic dual actions.

### Chemistry

### Synthetic route of the derivatives of bisindolediacetic acids

**Scheme 1:**
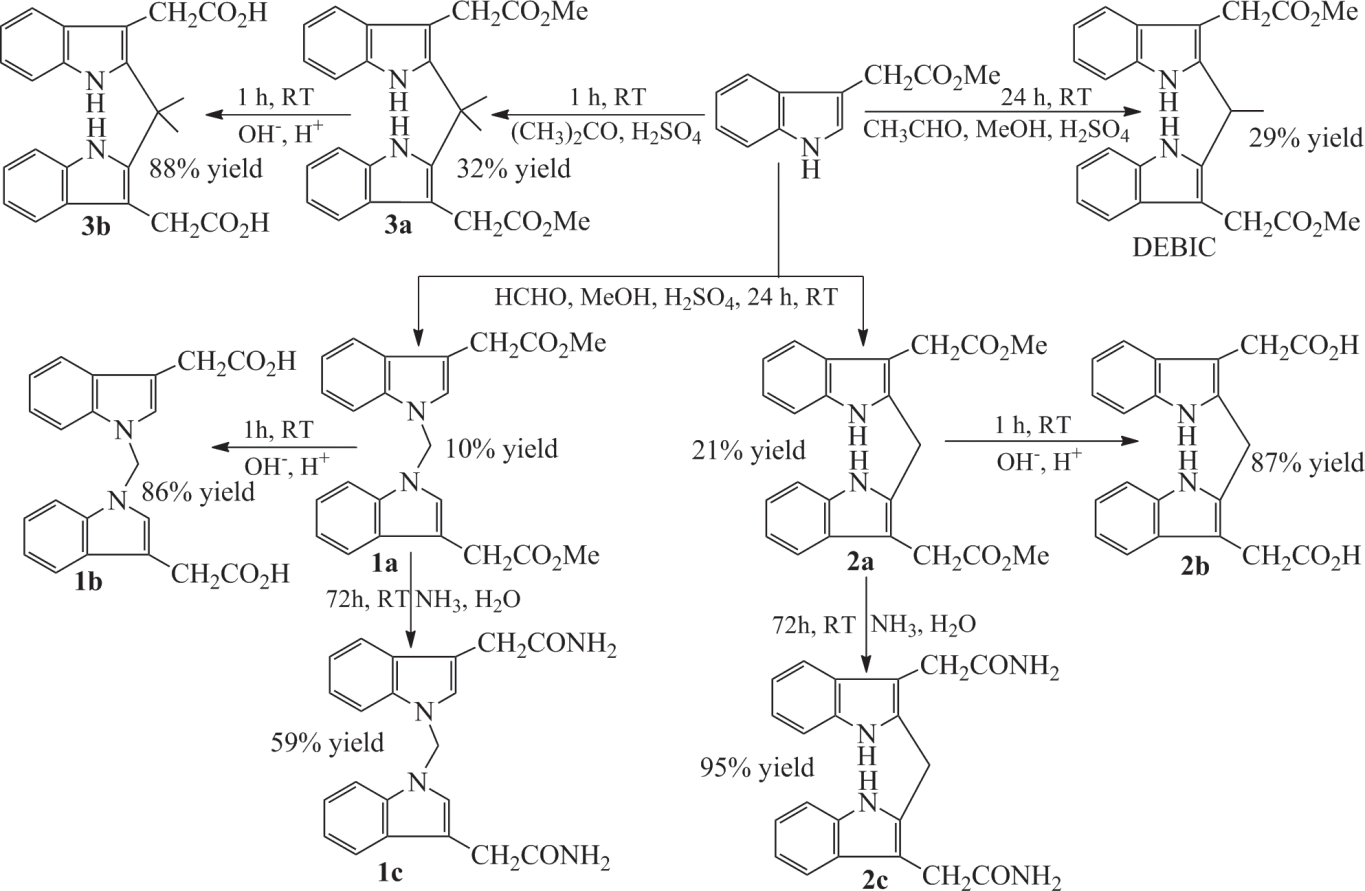
The synthetic route to the derivatives of bisindolediacetic acids, in which the reagents, conditions and yields are provided

The synthetic route of the derivatives of bisindolediacetic acids consists of three steps of Scheme 1. Briefly, at room temperature and in a pH 3 solution consisted of methanol, water and sulfuric acid, the methyl 1*H*-indole-3-ylacetate was condensed with formaldehyde for 24 h or with aldehyde for 24 h or with acetone for 1 h to provide dimethyl 2,2’-[1,1’-methylenebis(1*H*-indole-3,1-diyl)]diacetate (1a), dimethyl 2,2’-[(2,2’-methylenebis(1*H*-indole-3,2-diyl)]diacetate (2a), dimethyl 2,2’-[(2,2’- (propane-2,2-diyl)bis(1*H*-indole-3,2-diyl)]diacetate (3a) and DEBIC in 10%, 21%, 32% and 29% yield, respectively.

The hydrolysis of 1a, 2a and 3a was firstly carried out in a solution of NaOH in aqueous methanol at 0°C for 1 h to saponify, and then the reaction mixture was adjusted to pH 7.0 with hydrochloric acid to provide 2,2’-[1,1’-methylenebis(1*H*- indole-3,1-diyl)]diacetic acid (1b), 2,2’-[(2,2’-methylene)bis(1*H*-indole-3,2-diyl)]- diacetic acid (2b) and 2,2’-[(2,2’-(propane-2,2-diyl)bis(1*H*-indole-3,2-diyl)]diacetic acid (3b) in 87%, 86% and 88% yield, respectively.

The aminolysis of 1a, 2a and 3a was performed in a mixed solution of acetone and concentrated amonia water at room temperature for 72 h to provide 2,2’-[1,1’-methyle- nebis(1*H*-indole-3,1-diyl)]diaceamide (1c), 2,2’-[(2,2’-methylenebis(1*H*-indole-3,2- diyl)]diacetamide (2c) and 2,2’-[(2,2’-(propane-2,2-diyl)bis(1*H*-indole-3,2-diyl)]diace- tamide (3c) in 59%, 95% and 88% yield, respectively.

The mentioned procedure, reagents, conditions and yields demonstrate that these routes can successfully provide the designed derivatives of bisindolediacetic acids in a green chemical manner.

**Figure 2:**
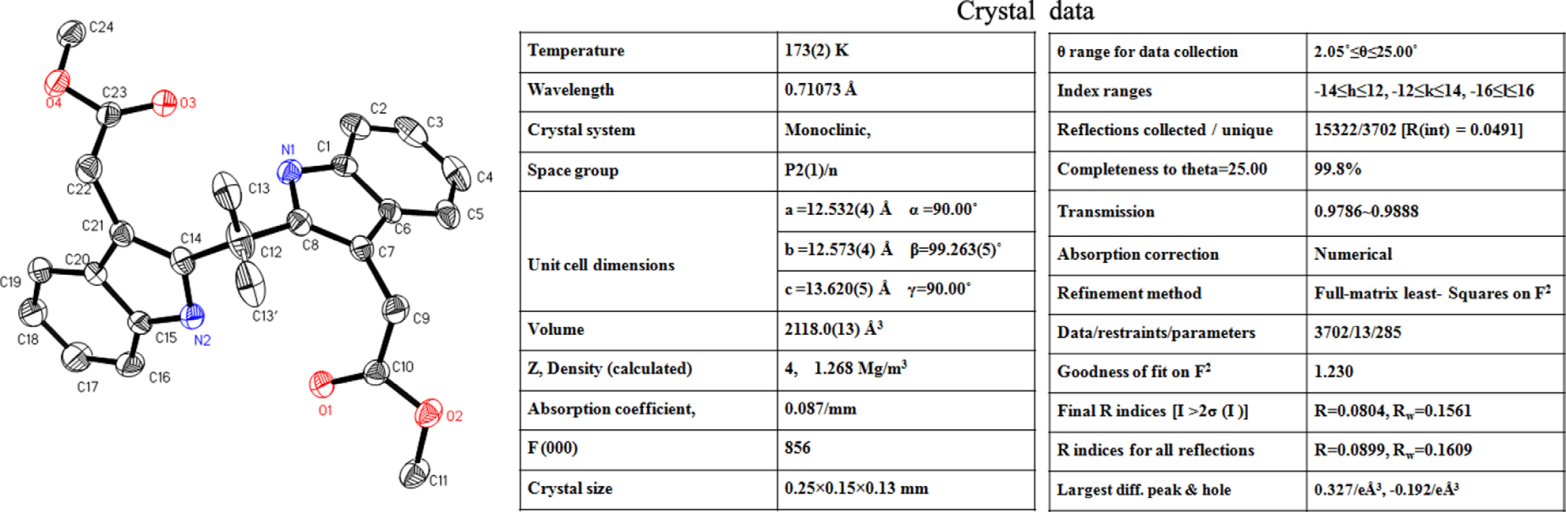
Crystal structure and corresponding parameter of DEBIC

As a part of chemistry the single crystal of the most active derivative DEBIC was prepared. The crystal structure and corresponding data of DEBIC are shown in Figure 2. Consequently, the conformation reflected by this crystal structure was used for the further investigations of DEBIC.

### Pharmacology and bioactivity

### Anti-tumor activity of nine derivatives of bisindolediacetic acids

**Figure 3:**
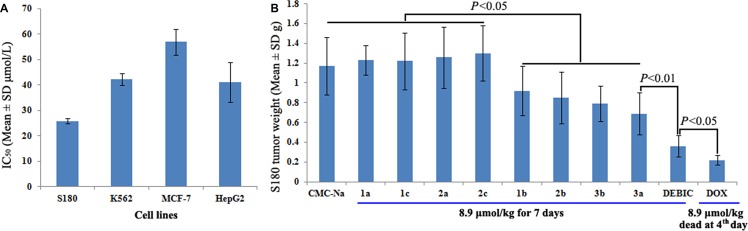
Results from the anti-tumor assays (**A**) IC_50_ of DEBIC against S180, K562, MCF-7 and HepG2 cells, *n* = 6; (**B**) *In vivo* anti-tumor activities of 9 derivatives of bisindolediacetic acids on S180 mouse model, *n* = 12.

To select an *in vivo* model for evaluating 9 derivatives of the bisindolediacetic acids, DEBIC was used as their representative to receive the *in vitro* anti-proliferation assay. Figure 3A gives the IC_50_ values of DEBIC against S180 (25.8 μM), K562 (42.27 μM), MCF-7 (56.92 μM) and HepG2 (41.1 μM), and shows that 25.8 μM of S180 cells is the lowest IC_50_ value. In this profile S180 tumor bearing mice were used as the *in vivo* model to evaluate the anti-tumor activities of 9 derivatives. Figure 3B shows that at 8.9 μmol/kg/day of oral injection dose for seven consecutive days. Five of the nine derivatives significantly slow tumor growth and DEBIC has the highest activity, while at 8.9 μmol/kg/day of i.p injection dose for four consecutive days doxorubicin (Dox) causes all mice to die. This assay suggests that DEBIC is the most promising lead compound and is worthy of the following investigations.

### Anti-tumor and anti-thrombotic activities of DEBIC

**Figure 4:**
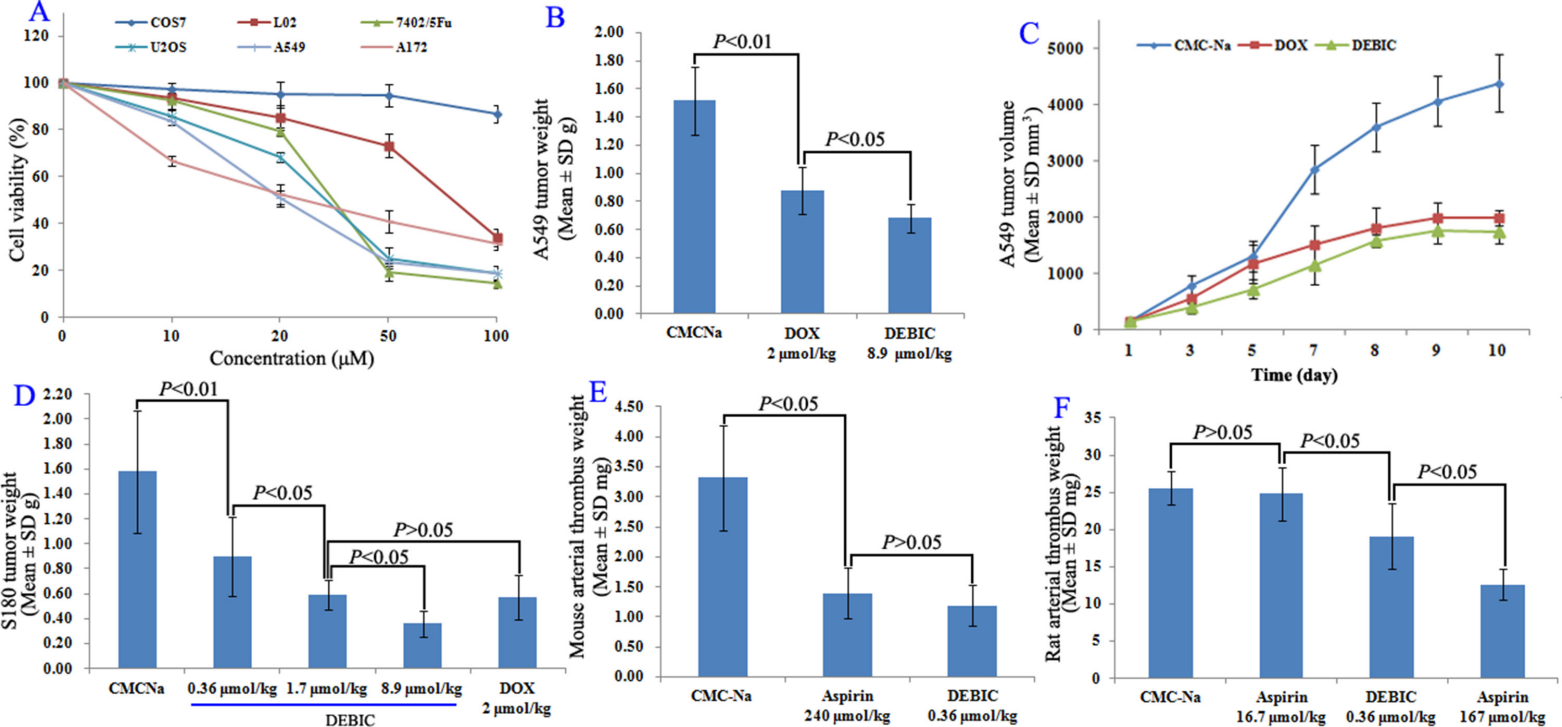
Anti-tumor and anti-thrombotic assays for DEBIC (**A**) Cell viabilities of DEBIC treated tumor cells Bel-7402/5Fu, U2OS, A172 and A549 cells as well as non-tumor cells COS7 and L02, *n* = 6; (**B**) Anti-tumor activity of oral DEBIC on A549 BABL/C mouse model for nine consecutive days, *n* = 12; (**C**) Tumor volume of DEBIC treated A549 BABL/C mice, *n* = 12; (**D**) Dose dependent anti-tumor activities of oral DEBIC on S180 mouse model for seven consecutive days, *n* = 12; (**E**) Anti-thrombotic activity of DEBIC on mouse model, *n* = 12; (**F**) Anti-thrombotic activity of DEBIC on rat model, *n* = 12.

The *in vitro* anti-proliferation assay of DEBIC was performed on both tumor and non-tumor cells. Figure 4A shows that the IC_50_ values of DEBIC against tumor cells Bel-7402/5Fu, U2OS, A172 and A549 are less than 30 μM, while the IC_50_ values of DEBIC against non-tumor cells COS7 and L02 are close or more than 100 μM. Due to A549 cells are the most sensitive tumor cells to DEBIC, the *in vivo* anti-tumor assay was additionally performed on A549 BABL/C mice. Figure 4B shows that at 8.9 μmol/kg/day for 9 consecutive days the oral DEBIC effectively slows tumor growth, and its activity is significantly higher than that of 2 μmol/kg/day of i.p injection Dox. A similar result is observed when the anti-tumor activity is represented with the tumor volume (Figure 4C).

The *in vivo* dose dependent anti-tumor action of DEBIC for seven consecutive days was performed on S180 mice. For this purpose the oral 0.36, 1.7 and 8.9 μmol/kg/day doses were used. Figure 4D shows that the tumor weight is gradually decreased when the oral dose is gradually increased, and the minimal effective dose is 0.36 μmol/kg/day.

The anti-arterial thrombosis activity was evaluated on both mouse and rat models, and represented with thrombus weight. Figure 4E shows that 0.36 μmol/kg of oral DEBIC effectively inhibits ferric chloride solution to trigger mouse abdominal aorta to form thrombus, the thrombus weight is significantly lower than that of the mice treated with carboxymethylcellulose sodium (CMC-Na), and is equal to that of the mice treated with 240 μmol/kg of oral aspirin. This means that the activity of DEBIC is equal to that of 666.7 folds of aspirin. Figure 4F shows that at 0.36 μmol/kg of oral dose DEBIC effectively inhibits the rats to form arterial thrombus, the thrombus weight is significantly lower than those of the rats treated with CMC-Na and with 16.7 μmol/kg of oral aspirin. This means that the activity of DEBIC is 46.4 folds higher than that of aspirin.

### DEBIC targeting P-selectin and intercalating CT DNA

**Figure 5:**
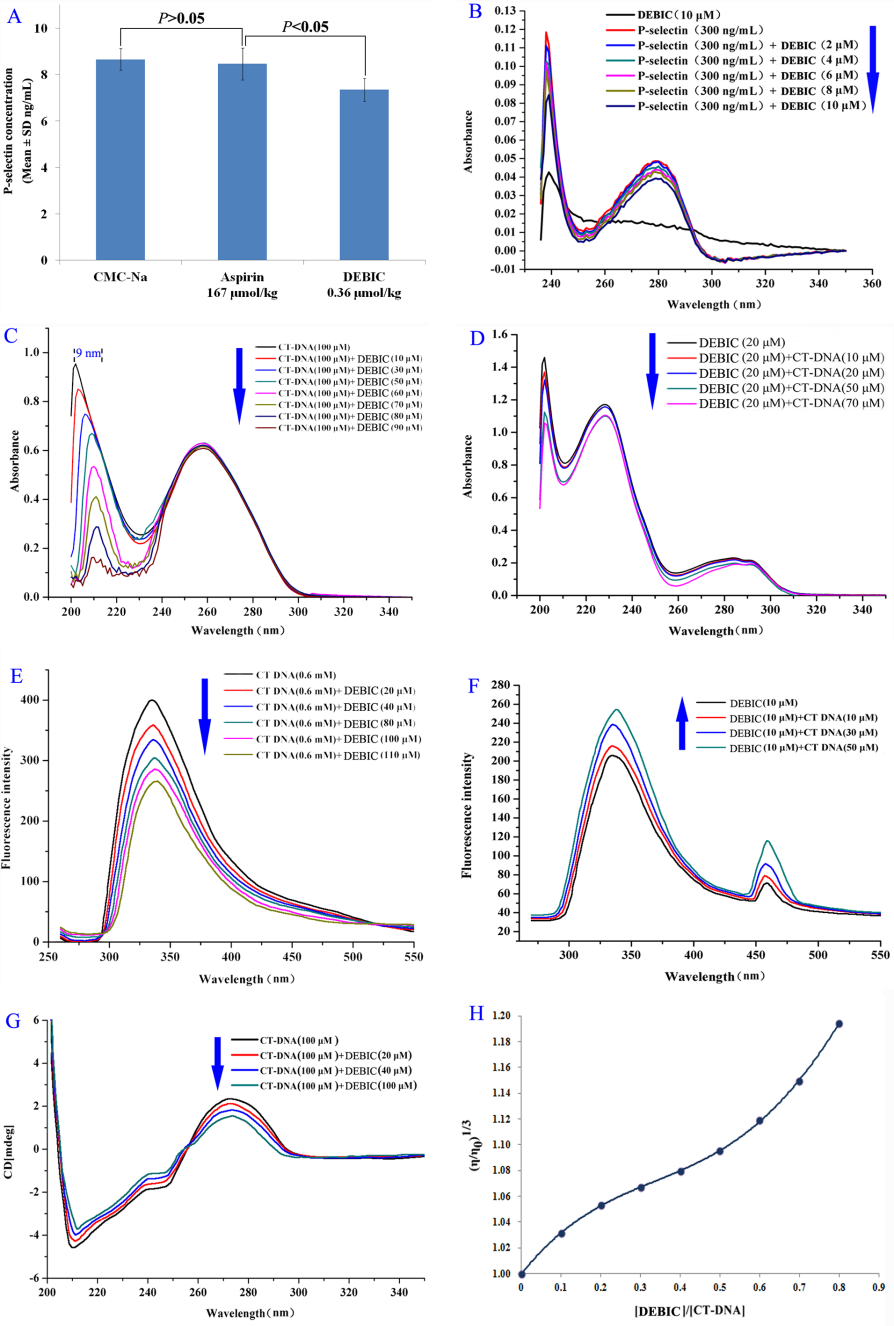
DEBIC targeting P-selectin and intercalating CT DNA (**A**) Concentration of soluble P-selectin in the serum of the arterial thrombosis rats treated with 0.36 μmol/kg of oral DEBIC; (**B**) Effect of DEBIC on the UV spectrum of soluble P-selectin; (**C**) Hypochromic effect and bathochromic shift occurred in the UV spectra of CT DNA (100 μM) plus DEBIC (0, 10, 30, 50, 60, 70, 80 and 90 μM); (**D**) Hypochromic effect occurred in the UV spectra of DEBIC (20 μM) plus CT DNA (0, 10, 20, 50 and 70 μM); (**E**) Concentration dependent course of fluorescence quenching of CT DNA (in pH 7.4 PBS, 600 μM) in the presence of DEBIC (0, 20, 40, 80, 100 and 110 μM); (**F**) Concentration dependent course of fluorescence quenching of DEBIC (in pH 7.4 PBS, 10 μM) in the presence of CT DNA (0, 10, 30 and 50 μM); (**G**) Effect of DEBIC on the CD spectra of CT DNA (in pH7.4 PBS, 100 μM); (**H**) Effect of DEBIC of various concentrations on the relative viscosity of CT DNA (37°C).

The targeting action of DEBIC on P-selectin was examined with the expression assay of P-selectin. Figure 5A shows the results of ELISA experiments, suggesting that the concentration of the soluble P-selectin in the serum of the arterial thrombosis rats treated with 0.36 μmol/kg of oral DEBIC is significantly lower than those of the arterial thrombosis rats treated with CMC-Na and 167 μmol/kg of oral aspirin. Figure 5B shows the results of DEBIC concentration dependently changing the UV spectra of soluble P-selectin, thereby reflects that by targeting soluble P-selectin DEBIC changes its conformation and consequently inhibits its pathological features.

The intercalating action of DEBIC on DNA was examined with the spectrum assay of CT DNA. Figure 5C indicates DEBIC (0–90 μM) concentration dependently causing the UV spectra of CT DNA (100 μM in PBS) to occur hypochromic effect and bathochromic shift, thereby reflects the change of CT DNA conformation induced by the intercalation of DEBIC. Vice versa CT DNA also (0–70 μM) concentration dependently causes the UV spectra of DEBIC (20 μM in PBS) to occur hypochromic effect and bathochromic shift, suggesting the change of CT DNA conformation induced by the intercalation of DEBIC (Figure 5D).

Figure 5E indicates DEBIC (0–110 μM) concentration dependently causing CT DNA (600 μM in PBS) to occur fluorescence quenching, i.e. decreasing fluorescence intensity of CT DNA, thereby reflects the intercalation of DEBIC to CT DNA. Vice versa CT DNA also (0–50 μM) concentration dependently causes DEBIC (10 μM in PBS) to occur fluorescence quenching, i.e. decreasing fluorescence intensity of DEBIC, suggesting the intercalation of DEBIC to CT DNA (Figure 5F).

Figure 5G indicates DEBIC (0–100 μM) concentration dependently causes the negative band (at ∼210 nm) and the positive band (at ∼275 nm) of circular dichroic (CD) spectra of CT DNA (100 μM in PBS) to increase and decrease the intensity, respectively. These band intensity changes can be attributed to the right-handed helicity (213 nm) and the base stacking (275 nm) been quite sensitive to the intercalation of DEBIC to CT DNA. Figure 5H indicates the relative viscosity of CT DNA been gradually increased with the increase of the concentration of DEBIC, suggesting to accommodate and bind DEBIC for performing the intercalation the base pairs of CT DNA are pushed apart and consequently results in the increase of the viscosity.

### DEBIC inhibiting platelet aggregation

**Figure 6:**
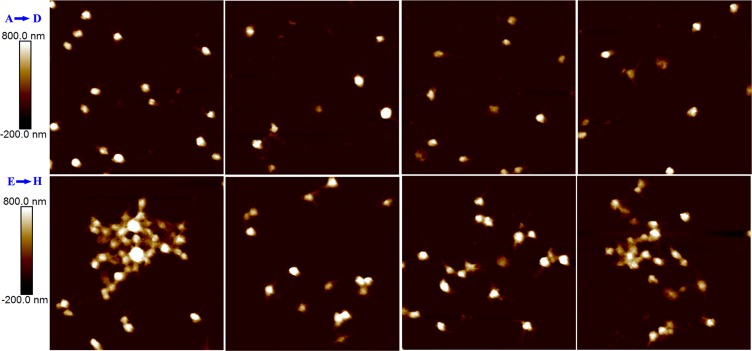
AFM visualized the effect of DEBIC on the resting rat platelets and AA activated rat platelets (**A**) AFM image of resting rat platelets; (**B**) AFM image of resting rat platelets with DEBIC (10^-2^ mg/L); (**C**) AFM image of resting rat platelets with DEBIC (10^-4^ mg/L); (**D**) AFM image of resting rat platelets with DEBIC (10^-6^ mg/L); (**E**) AFM image of AA activated rat platelets; (**F**) AFM image of AA activated rat platelets with DEBIC (10^-2^ mg/L); (**G**) AFM image of AA activated rat platelets with DEBIC (10^-4^ mg/L); (**H**) AFM image of AA activated rat platelets with DEBIC (10^-6^ mg/L).

The role of platelet activation in tumor growth and thrombosis are well known [24, 25]. To visualize the effect of DEBIC on platelet activation the resting rat platelets and arachidonic acid (AA) activated rat platelets were treated with DEBIC to record their atomic force microscopy (AFM) images. Figure 6A visualizes that the resting platelets have no pseudopodia. Figure 6B–6D visualize that DEBIC (10^−2^ mg/L-10^−6^ mg/L) does not change the morphology of the resting platelets, and the platelets still have no pseudopodia. Figure 6E visualizes that AA (0.4 mM) activation induces rat platelets to extend their pseudopodia and to form aggregation. Figure 6F, 6G and 6H visualize that when the concentration of DEBIC is decreased from 10^−2^ mg/L to 10^−4^ mg/L and 10^−6^ mg/L the number of the extended pseudopodia and the aggregation level of AA activated rat platelets are gradually increased. DEBIC concentration dependently inhibits the activation of rat platelets. Thus the inhibition of platelet activation should be responsible for DEBIC slowing tumor growth and decreasing thrombus weight.

### DEBIC targeting thrombus

**Figure 7:**
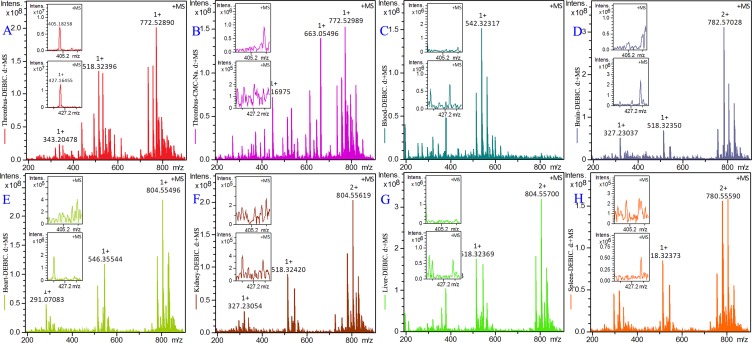
ESI(+)-FT-ICR-MS spectra of the extracts of thrombus, blood, brain, heart, kidney, liver and spleen of the treated thrombosis rats, *n* = 12 (**A**) ESI(+)-FT-ICR-MS spectrum of the extract of the thrombus of 0.36 μmol/kg oral DEBIC treated rats; (**B**) ESI(+)-FT-MS spectrum of the extract of the thrombus of CMC-Na treated rats; (**C**–**H**) ESI (+)-FT-ICR-MS spectra of the extracts of the blood, brain, heart, kidney, liver and spleen of 0.36 μmol/kg oral DEBIC treated rats.

To demonstrate the targeting action of DEBIC the thrombus, blood, brain, heart, kidney, liver and spleen of the rats treated by 0.36 μmol/kg of oral DEBIC and the thrombus of the rats treated by CMC-Na were homogenized, the homogenates were extracted with methanol and the extracts were received ESI(+)-FT-ICR-MS analysis. The ESI(+)-FT -ICR-MS spectrum of the thrombus extract of 0.36 μmol/kg oral DEBIC treated rats gives an ion peak at 405.18258, the mass of DEBIC plus H, and an ion peak at 427.16455, the mass of DEBIC plus Na (Figure 7A). These ion peaks mean that in the thrombus there is DEBIC. The ESI(+)-FT-ICR- MS spectrum of the extract of the thrombus of CMC-Na treated rats gives no any ion peak related to DEBIC (Figure 7B). Similarly, the ESI(+)-FT -ICR-MS spectra of the extracts of the blood, brain, heart, kidney, liver and spleen of 0.36 μmol/kg oral DEBIC treated rats also give no ion peak related to DEBIC (Figure 7C–7H). Therefore, all figures consistently evidence that DEBIC is able to target thrombus.

## DISCUSSION

At room temperature with sulfuric acid as the catalyst the simple condensation of methyl 1*H*-indole-3-yl-acetate and formaldehyde, aldehyde or acetone successfully formed the desirable dimethyl bisindolediacetate (1a-3a and DEBIC). Of them DEBIC was additionally characterized by its crystal structure. The hydrolysis and aminolysis of 1a-3a smoothly resulted in the bisindolediacetic acids (1b-3b) and the bisindolediacetamides (1c-3c), respectively. Therefore the synthetic route of Scheme 1 is suitable for the preparations.

To predict the possibility of 12 bisindolediacetic acid derivatives to target both P-selectin and d(CGATCG)_2_ their docking scores were calculated and found falling within a range of 31.9–55.6. To examine the reliability of docking investigation the anti-tumor activities of nine derivatives that the scores fell within a range of 38.0–55.6 were evaluated with S180 mice. It was found that the docking scores of 1b, 2b, 3a, 3b and DEBIC fell within a range of 42–55 and the tumor weights of the mice treated with them were significantly lowered. Besides, the docking of DEBIC towards both P-selectin and d(CGATCG)_2_ gave the highest scores, the mice treated by it had the smallest tumor and the rats treated by it had the smallest thrombus. The correlation suggests that the docking investigation could be suitably used to predict the anti-tumor and anti-thrombotic activities of the derivatives of bisindolediacetic acids, and DEBIC could be a lead compound for further anti-tumor and the anti-thrombotic evaluations.

The anti-tumor evaluations include the *in vitro* anti-proliferation of DEBIC against tumor cells and non-tumor cells, as well as the *in vivo* action of DEBIC on A549 BABL/C and S180 mice. The fact that the IC_50_ values of DEBIC against tumor cells are less than 30 μM, while the IC_50_ values of DEBIC against non-tumor cells are close or more than 100 μM means DEBIC injures tumor cells, but not non-tumor cells, and should be a safe anti-tumor agent (Figure 4A). This was supported by the *in vivo* anti-tumor assay. On S180 mouse model, the minimal effective dose of seven consecutive days is 0.36 μmol/kg/day, but even at a dose of 8.9 μmol/kg/day for seven consecutive days DEBIC still induces no mouse to die (Figure 4D). In contrast, at a dose of 8.9 μmol/kg/day for only four consecutive days Dox induces all mice to die (Figure 3B). The *in vivo* anti-tumor efficacy of DEBIC was further examined on A549 BABL/C mouse model. Even at an oral dose of 8.9 μmol/kg/day for nine consecutive days DEBIC effectively slows tumor growth and induces no any mouse to die. On the other hand, only at an i.p injection dose of 2 μmol/kg/day for nine consecutive days Dox induces no any mouse to die, but the activity is significantly lower than that of 8.9 μmol/kg/day of oral DEBIC (Figure 4B). A similar result is observed when the anti-tumor activity is represented with tumor volume (Figure 4C). These comparisons emphasize both the safety and effectiveness of oral DEBIC.

The anti-thrombotic evaluation on mouse model shows that at 0.36 μmol/kg of oral dose the activities of DEBIC inhibiting mouse arterial thrombosis is equal to 240 μmol/kg of oral aspirin, and on rat models shows that at 0.36 μmol/kg of oral dose the activities of DEBIC inhibiting rat arterial thrombosis is equal to 167 μmol/kg and higher than 16.7 μmol/kg of oral aspirin, respectively. Platelet activation may simultaneously promote both arterial thrombosis and tumor growth [22–25], and here were correlated with AFM images. AFM image visualizes that both the aggregation level and the extended amount of the pseudopodia of AA activated platelets are decreased by DEBIC in a concentration dependent manner. As depicted by Figure 6, the visual impression of AFM assay is more direct than the anti-platelet aggregation assay in the understanding of the impact of DEBIC on platelet activation.

Here the advantages of DEBIC in both anti-tumor and anti-thrombotic actions are shown by DEBIC docking into the active site of P-selectin, by DEBIC changing UV based conformation of soluble P-selectin and by DEBIC depressing soluble P-selectin level in the serum of the treated thrombosis rats.

Additionally, the intercalation of DEBIC to CT DNA may alter the conformation or the spatial relation of the double α-helix of CT DNA, and these alterations could be visualized by UV, fluorescence and CD spectra as well as the relative viscosity of the CT DNA. Thus the concentration dependent effects of DEBIC on the UV spectra, the fluorescent spectra, the CD spectra and the relative viscosity of CT DNA were used to visualize the anti-tumor mechanism of DEBIC.

Finally, the ESI(+)-FT-ICR-MS spectra of the extracts of the thrombus, the blood and the organs of the rats orally treated with 0.36 μmol/kg DEBIC and the ESI(+)-FT-ICR-MS spectrum of the extracts of the thrombus of the rats orally treated with CMC-Na were compared. The comparison revealed that only the ESI(+)-FT-ICR-MS spectrum of the thrombus extract of DEBIC treated rats gave an ion peak of DEBIC. Thus ESI(+)- FT-ICR-MS analysis ensured a targeting action of DEBIC on the thrombus, experimentally evidenced the rationality of DEBIC having the highest score in docking the active site of P-selectin, and agreed the down-regulation of P-selectin expression *in vivo*.

## MATERIALS AND METHODS

### General

All the reactions were carried out under nitrogen (1 bar). ^1^H (300 and 800 MHz) and ^13^C (75 and 200 MHz) NMR spectra were recorded on Bruker Avance-300 and Avance-III- 800 spectrometers for solution DMSO-*d*_6_ with tetramethylsilane as internal standard. IR spectra were recorded with a Perkin-Elmer 983 instrument. ESI(+)-FT-ICR-MS were recorded on a 9.4 T solariX Fourier transform ion cyclotron resonance mass spectrometer (Bruker Corp, Billerica, MA, USA). Melting points were measured on a XT5 hot stage microscope (Beijing key electro-optic factory). TLC was made with Qingdao silica gel GF_254_. Chromatography was performed with Qingdao silica gel H_60_ or Sephadex-LH_20_. All solvents were distilled and dried before use by following literature procedures. HPLC purities (Waters, C_18_ column 4.6 × 150 mm) of all products ranged from 98.0% to 98.9%. An Agilent Technologies 1200 Series HPLC system (Agilent Technologies, Santa Clara, CA, USA) was used. The derivatives were separated on a Waters XTerra C_18_ reversed phase column (2.1 × 150 mm, 5 μm; Waters Limited, Hertfordshire, UK) protected by a guard column of the same material (5 × 10 mm, 5 μm). The column thermostat was maintained at 40°C. To the column, 5 μL solution of the derivatives in ultrapure water was injected for analysis. The mobile phase consisted of water and acetonitrile (5/95). The flow rate was 0.2 mL/min. The column was washed with water and methanol (35/65), and equilibrated to initial conditions for 15 min. Ultraviolet (UV) absorption spectra were recorded online. The UV detector was set to a scanning range of 200–400 nm, and a wavelength of 254 nm was used to monitor the chromatograms of the derivatives.

Male Sprague Dawley rats (200–250 g) and male ICR mice (20–22 g) were purchased from the Laboratory Animal Center of Capital Medical University. The protocol of all evaluations was reviewed and approved by Ethics Committee of Capital Medical University. The committee assured that the animal welfare was maintained in accordance to the requirements of Animal Welfare Act and NIH Guide for Care and Use of Laboratory Animals. Statistical analyses of all biological data were carried out by use of ANOVA, and LSD for multiple group comparison. All analyses were done with SPSS 19.0 program, and *P*-value < 0.05 was considered statistically significant.

### Generation of conformation for docking

The 2D structure of 12 derivatives of bisindole-2-carboxilic acids was sketched in ChemDraw Ultra 10.0, converted to conformation in Chem3D 10.0, and then energy minimized in Discovery Studio 3.5 with a Merck molecular force field (Merck & Co.) until the minimum RMS reached 0.001 in Chem3D Ultra 10.0. The energy optimized conformations in whole conformational space of the derivatives were sampled with systematic search and BEST method of Discovery Studio 3.5, which were practiced with a SMART minimizer using CHARMM force field. The energy threshold was set to 20 kcal/mol at 300 K. The maximum minimization steps were 200 and the minimization root mean squared (RMS) gradient was 0.1 Å. The maximum generated conformations were 255 with a RMS deviation (RMSD) cutoff of 0.2 Å. Top 10 energy optimized conformations of DEBIC were used for the docking to d(CGATCG)_2_ and P-selectin.

### Docking toward active site of d(CGATCG)_2_ or P-selectin

Software AutoDock 4 was used to perform the docking of 10 energy optimized conformations of the derivatives toward d(CGATCG)_2_ or P-selectin. d(CGATCG)_2_ or P-selectin was treated as rigid and prepared by AutoDockTools 1.5, i.e. merging nonpolar hydrogens and assigning gasteiger charges and autodock elements. The 10 energy optimized conformations of the derivatives were treated as rigid ligands and prepared by AutoDockTools 1.5 i.e. merging nonpolar hydrogens, assigning gasteiger charges, finding root and aromatic carbons, detecting rotatable bonds, and setting torsions. The grid box dimensions were set to 50 Å × 50 Å × 50 Å with a grid spacing of 0.375 Å. Lamarckian genetic algorithm (LGA) was used to find the appropriate binding positions, orientations, and the conformations of the derivatives in the active site pocket of d(CGATCG)_2_ or P-selectin. The global optimization was started with parameters of a population of 300 randomly positioned individuals. The maximum number of energy evaluations was increased to 2.5 × 10^7^, and the maximum number of generations in the LGA algorithm was increased to 2.7 × 10^5^. The Solis and Wets local search was performed with a maximum number of 3000. During the molecular docking experiments, 200 runs were carried out for each ligand. The resulted 200 conformations of each ligand were scored by the lowest binding energy and clustered with an rms tolerance of 2.0 Å.

### Synthesis

### Dimethyl 2,2’-[1,1’-methylenebis(1*H*-indole-3,1-diyl)]diacetate (1a)

At room temperature the solution of 1.0 g (5.7 mmol) of 1*H*-indole-3-yl-acetic acid, 20 mL of aqueous sulfuric acid and 2 mL of formaldehyde (40%) was stirred at room temperature for 24 h and TLC (ethyl acetate: petroleum ether, 1:2) indicated complete disappearance of (1*H*-indole-3-yl)-acetic acid. The reaction mixture was adjusted to pH 3 with concentrated sulfuric acid and the formed precipitates were collected by filtration. At 0°C the solution of 2.5 mL of SOCl_2_ and 20 mL of methanol was mixed with the collected precipitates. This mixture was stirred at 0°C for 30 min and at room temperature for 4 h. The reaction mixture was evaporated under vacuum. The residue was dissolved in 50 mL of ethyl acetate, the formed solution was washed successively with saturated aqueous solution of NaHCO_3_ (30 mL × 3), 5% aqueous solution of KHSO_4_ (30 mL × 3) and saturated aqueous solution of NaCl (30 mL × 3) and the ethyl acetate phase was dried with anhydrous Na_2_SO_4_. After filtration the filtrate was evaporated under vacuum and the residue was separated on silica gel column (ethyl acetate: petroleum ether, 1: 4) to provide 240 mg (21%) of the title compound as colorless powders. Mp 110–112°C. IR (KBr, cm^-1^): 3059, 2953, 2926, 2843, 2361, 2342, 1732, 1655, 1560, 1458, 1437, 1395, 1350, 1323, 1298, 1275, 1261, 1244, 1198, 1142, 1101, 1038, 1011, 939, 827, 800, 777, 750, 698, 596, 553, 530, 453, 433. ^1^H NMR (300 MHz, DMSO-*d6*): δ/ppm = 7.81 (d, *J* = 8.4 Hz, 2H), 7.59 (s 2H), 7.47 (d, *J* = 7.5 Hz, 2H), 7.20 (t, *J* = 7.5 Hz, 2H), 7.05 (t, *J* = 7.5 Hz, 2H), 6.60 (s, 2H), 3.73 (s, 4H), 3.62 (s, 6H). ^13^C NMR (75 MHz, DMSO-*d6*): δ/ppm = 172.2, 136.1, 128.3, 127.8, 122.4, 120.0, 119.4, 110.7, 108.6, 55.4, 52.0, 30.7. ESI(+)-MS (m/z): 391 [M + H]^+^. Anal. Calcd for C_23_H_22_N_2_O_4_: C, 71.75; H, 6.26; N, 6.69. Found C, 71.53; H, 6.10; N, 6.90.

### Dimethyl 2,2’-[(2,2’-methylenebis(1*H*-indole-3,2-diyl)]diacetate (2a)

By using the procedure of preparing 1a 330 mg (29%) of the title compound was obtained as colorless powders. Mp 79–81°C. IR (KBr, cm^-1^): 3267, 3211, 3057, 3032, 3007, 2951, 2916, 2839, 2750, 2610, 2361, 2342, 1910, 1871, 1728, 1707, 1676, 1624, 1589, 1491, 1460, 1433, 1418, 1350, 1327, 1302, 1294, 1231, 1186, 1163, 1134, 1105, 1009, 991, 974, 849, 799, 737, 669, 650, 629, 588, 550, 536. ^1^H NMR (300 MHz, DMSO-*d6*): δ/ppm = 10.73 (s, 2H), 7.41 (d, *J* = 7.5 Hz, 2H), 7.28 (d, *J* = 7.5 Hz, 2H), 7.03 (t, *J* = 6.9 Hz, 2H), 6.97 (t, *J* = 6.9 Hz, 2H), 4.26 (s, 2H), 3.76 (s, 4H), 3.56 (s, 6H). ^13^C NMR (75 MHz, DMSO-*d6*): δ/ppm = 172.7, 135.8, 134.1, 128.5, 121.2, 119.1, 118.3, 111.3, 104.5, 51.9, 29.8, 23.5. ESI(+)-MS (m/z): 391 [M + H]^+^. Anal. Calcd for C_23_H_22_N_2_O_4_: C, 70.75; H, 5.68; N, 7.17. Found C, 70.94; H, 5.51; N, 7.39.

### Dimethyl 2,2’-[(2,2’-(propane-2,2-diyl)bis(1*H*-indole-3,2-diyl)]diacetate (3a)

At room temperature the solution of 1.0 g (5.3 mmol) of 1*H*-indol-3-yl-acetic acid methyl ester, 10 mL of acetone and 1 mL of concentrated sulfuric acid was stirred at room temperature for 1 h and TLC (ethyl acetate: petroleum ether, 1:3) indicated the complete disappearance of 1*H*-indol-3-yl-acetic acid methyl ester. The reaction mixture was adjusted to pH 7 with aqueous sodium hydroxide (2 M) and then evaporated under vacuum. The residue was dissolved in 50 mL of ethyl acetate, the formed solution was washed successively with saturated aqueous solution of NaHCO_3_ (30 mL × 3), 5% aqueous solution of KHSO_4_ (30 mL × 3) and saturated aqueous solution of NaCl (30 mL × 3) and dried with anhydrous Na_2_SO_4_. After filtration the filtrate was evaporated under vacuum and the residue was separated on silica gel column to provide 370 mg (32%) of the title compound. Mp. 156–158°C. IR (KBr, cm^-1^): 3383, 3323, 3059, 3024, 3005, 2976, 2951, 2361, 2342, 1713, 1622, 1560, 1508, 1497, 1462, 1437, 1420, 1323, 1300, 1234, 1201, 1182, 1165, 1142, 1121, 1013, 993, 790, 744, 711, 669, 624, 611, 588. ^1^H NMR (300 MHz, DMSO-*d6*): δ/ppm = 10.84 (s, 2H), 7.38 (d, *J* = 8.1 Hz, 2H), 7.31 (d, *J* = 7.8 Hz, 2H), 7.05 (t, *J* = 7.5 Hz, 2H), 6.94 (t, *J* = 7.5 Hz, 2H), 3.34 (s, 3H), 3.30 (s, 3H), 3.14 (s, 4H), 1.80 (s, 6H). ^13^C NMR (75 MHz, DMSO-*d6*): δ/ppm = 172.2, 142.2, 135.0, 129.5, 121.0, 119.0, 118.3, 111.6, 103.6, 51.5, 33.9, 29.5, 23.3. ESI(+)-MS (m/z): (m/z): 419 [M + H]^+^. Anal. Calcd for C_25_H_26_N_2_O_4_: C, 71.75; H, 6.26; N, 6.69; O, 15.29. Found C, 71.54; H, 6.11; N, 6.91.

### Dimethyl 2,2’-(2,2’-(ethane-1,1-diyl)bis(1H-indole-3,2-diyl))diacetate (DEBIC)

To a solution of 1 g (5.7 mmol) of 1*H*-indole-3-yl-acetic acid methyl ester in 30 mL of methanol 0.5 mL of concentrated sulphuric acid was added dropwise. To this solution 2 mL of ethyl aldehyde (40%) was added, the reaction mixture was stirred at room temperature for 24 h and evaporated under vacuum. The residue was dissolved in 50 mL of ethyl acetate, the formed solution was washed successively with saturated aqueous solution of NaHCO_3_ (30 mL × 3), 5% aqueous solution of KHSO_4_ (30 mL × 3) and saturated aqueous solution of NaCl (30 mL × 3) and dried with anhydrous Na_2_SO_4_. After filtration the filtrate was evaporated under vacuum and the residue was purified on silica gel column (ethyl acetate: petroleum ether, 1:4) to provide 320 mg (28%) of the title compound as colorless powders. Mp 162–164°C. IR (KBr, cm^-1^): 3296, 2976, 2953, 2842, 1709, 1623, 1590, 1565, 1493, 1460, 1434, 1416, 1374, 1325, 1314, 1281, 1228, 1201, 1170, 1139, 1107, 1076, 1059, 992, 974, 741, 693, 679. ^1^HNMR (800 MHz, DMSO-*d6*): δ/ppm = 10.81 (s, 2H), 7.39 (d, *J* = 8.0 Hz, 2H), 7.35 (d, *J* = 8.0 Hz, 2H), 7.05 (t, *J* = 8.0 Hz, 2H), 6.96 (t, *J* = 8.0 Hz, 2H), 4.77 (q, *J* = 7.2 Hz, 1H), 3.65 (q, *J* = 8.0 Hz, 4H), 3.49 (s, 6H), 1.73 (d, *J* = 7.2 Hz, 3H). ^13^C NMR (200 MHz, DMSO-*d6*): δ/ppm = 172.4, 138.6, 135.7, 128.4, 121.1, 119.1, 118.4, 111.5, 103.5, 51.9, 29.7, 29.1, 20.2. ESI(+)-MS (m/z): 405 [M + H]^+^. Anal. Calcd for C_24_H_24_N_2_O_4_: C, 71.27; H, 5.98; N, 6.93. Found C, 71.08; H, 5.82; N, 6.70.

### 2,2’-[1,1’-Methylenebis(1*H*-indole-3,1-diyl)]diacetic acid (1b)

A solution of 50 mg (0.13 mmol) of 1a in 0.5 mL of methanol was adjusted pH 12 by adding aqueous NaOH (2M) dropwise. This solution was stirred at room temperature for 2 h and TLC (ethyl acetate: petroleum ether, 1:2) indicated the complete disappearance of 1a. At room temperature the reaction mixture was evaporated under vacuum to remove methanol, the residue was adjusted to pH 2 with hydrochloric acid, and extracted with ethyl acetate (30 mL × 3). The ethyl acetate phase was dried with anhydrous Na_2_SO_4_, filtered and the filtrate was evaporated under vacuum to give 41 mg (88%) the title compound as colorless powders. Mp 154–156°C. IR (KBr, cm^-1^): 3393, 3053, 3083, 2924, 2556, 2361, 2342, 1707, 1654, 1618, 1560, 1460, 1406, 1350, 1329, 1321, 1271, 1229, 1204, 1153, 1040, 1016, 932, 795, 743, 669, 586, 426. ^1^H NMR (300 MHz, DMSO-*d6*): δ/ppm = 12.08 (s, 2H), 7.81 (d, *J* = 8.1 Hz, 2H), 7.55 (s 2H), 7.48 (d, *J* = 8.1 Hz, 2H), 7.19 (t, *J* = 7.5 Hz, 2H), 7.04 (t, *J* = 7.5 Hz, 2H), 6.60 (s, 2H), 3.61 (s, 4H). ^13^C NMR (75 MHz, DMSO-*d6*): δ/ppm = 173.2, 136.1, 128.4, 127.6, 122.3, 119.8, 119.5, 110.6, 109.2, 55.4, 31.2. ESI(-)-MS (m/z): 361 [M-H]^-^. Anal. Calcd for C_21_H_18_N_2_O_4_: C, 69.60; H, 5.01; N, 7.73; O, 17.66. Found C, 69.79; H, 5.16; N, 7.92.

### 2,2’-[(2,2’-Methylenebis(1*H*-indole-3,2-diyl)]diacetic acid (2b)

By using the procedure of preparing 1b from 50 mg (0.13 mmol) of 2a 37 mg (80%) of the title compound was obtained as colorless powders. Mp 205–207°C. IR (KBr, cm^-1^): 3393, 3356, 3337, 3113, 3057, 3038, 3022, 3005, 2978, 2958, 2926, 2855, 2607, 2573, 2361, 2342, 1701, 1692, 1655, 1624, 1560, 1508, 1491, 1460, 1439, 1412, 1341, 1321, 1305, 1277, 1234, 1180, 1132, 1103, 745, 669. ^1^H NMR (300 MHz, DMSO-*d6*): δ/ppm = 12.29 (s, 2H),10.66 (s, 2H) 7.43 (d, *J* = 7.5 Hz, 2H), 7.23 (d, *J* = 7.5 Hz, 2H), 7.02 (t, *J* = 7.5 Hz, 2H), 6.99 (t, *J* = 7.5 Hz, 2H), 4.25 (s, 2H), 3.73 (s, 4H). ^13^C NMR (75 MHz, DMSO-*d6*): δ/ppm = 174.3, 135.8, 134.3, 128.5, 121.2, 119.0, 118.4, 111.2, 105.1, 30.2, 23.3. ESI(-)-MS (m/z): 361 [M-H]^-^. Anal. Calcd for C_21_H_18_N_2_O_4_: C, 69.60; H, 5.01; N, 7.73; O, 17.66. Found C, 69.41; H, 4.86; N, 7.50.

### 2,2’-[(2,2’-(Propane-2,2-diyl)bis(1*H*-indole-3,2-diyl)]diacetate (3b)

By using the procedure of preparing 1b from 100 mg (0.24 mmol) of 3a 57 mg (61%) of the title compound was obtained as colorless powders. Mp.160–162°C. IR(KBr, cm^-1^): 3410, 3379, 3356, 3059, 2980, 2899, 2361, 2341, 1701, 1655, 1638, 1560, 1508, 1491, 1458, 1340, 1310, 1246, 1222, 1206, 1163, 750, 721, 669. ^1^H NMR (300 MHz, DMSO-*d6*): δ/ppm = 11.92 (s 2H), 10.77 (s 2H), 7.37 (d, *J* = 7.8 Hz, 2H), 7.32 (d, *J* = 7.8 Hz, 2H), 7.04 (t, *J* = 7.8 Hz, 2H), 6.94 (t, *J* = 7.8 Hz, 2H), 3.09 (s, 4H). 1.82 (s, 6H). ^13^C NMR (75 MHz, DMSO-*d6*): δ/ppm = 173.7, 142.0, 135.0, 129.6, 120.9, 118.9, 118.5, 111.5, 104.2, 35.3, 30.3, 23.7. ESI(-)-MS (m/z): 389 [M-H]^-^. Anal. Calcd for C_23_H_22_N_2_O_4_: C, 70.75; H, 5.68; N, 7.17; O, 16.39 Found C, 70.96; H, 5.84; N, 7.40.

### 2,2’-[1,1’-Methylenebis(1*H*-indole-3,1-diyl)]diacetamide (1c)

To a solution of 100 mg (0.26 mmol) of 1a in 2 mL of acetone, 4 mL of concentrated amonia water was added dropwise. The reaction mixture was stirred at room temperature for 72 h and TLC (ethyl acetate: petroleum ether, 1:2) indicated complete disappearance of 1a. On removal of acetone the formed precipitates were collected by filtration to provide 54 mg (59%) of the title compound as colorless powders. Mp 226–228°C. IR (KBr, cm^-1^): 3433, 3374, 3356, 3337, 3198, 3057, 2924, 2855, 2361, 2342, 1655, 1649, 1611, 1560, 1508, 1458, 1317, 1283, 1269, 1202, 1157, 1121, 1101, 1038, 1016, 791, 740, 669, 569, 426. ^1^H NMR (300 MHz, DMSO-*d6*): δ/ppm = 7.80 (d, *J* = 8.4 Hz, 2H), 7.56 (s, 2H), 7.48 (d, *J* = 7.5Hz, 2H), 7.19 (t, *J* = 7.5 Hz 2H), 7.0 (d, *J* = 7.5Hz, 2H), 6.50 (s, 2H), 5.47 (s, 4H), 3.61 (s, 4H). ^13^C NMR (75 MHz, DMSO-*d6*): δ/ppm = 165.9, 136.1, 128.3, 127.8, 122.4, 119.4, 110.7, 108.6, 55.4, 52.0, 30.7. ESI(+)-MS (m/z): 361 [M + H]^+^. Anal. Calcd for C_21_H_20_N_4_O_2_: C, 70.75; H, 5.68; N, 7.17. Found C, 70.54; H, 5.53; N, 7.38.

### 2,2’-[(2,2’-Methylenebis(1*H*-indole-3,2-diyl)]diacetamide (2c)

By using the procedure of preparing 1c from 100 mg (0.26 mmol) of 2a 88 mg (95%) of the title compound was obtained as colorless powders. Mp 205°C. IR (KBr, cm^-1^): 3393, 3356, 3337, 3113, 3057, 3038, 3022, 3005, 2978, 2958, 2926, 2855, 2607, 2573, 2361, 2342, 1701, 1692, 1655, 1624, 1560, 1508, 1491, 1460, 1439, 1412, 1341, 1321, 1305, 1277, 1234, 1180, 1132, 1103, 745, 669. ^1^H NMR (300 MHz, DMSO-*d6*): δ/ppm = 11.72 (s, 2H), 7.40 (d, *J* = 7.8 Hz, 2H) 7.27 (d, *J* = 7.8 Hz, 2H), 7.03 (t, *J* = 7.8 Hz, 2H), 6.96 (t, *J* = 7.8 Hz, 2H), 5.47 (s, 4H), 4.25 (s, 2H), 3.75 (s, 4H). ^13^C NMR (75 MHz, DMSO-*d6*): δ/ppm = 167.6, 135.7, 134.8, 128.4, 121.2, 118.7, 118.3, 110.9, 105.5, 31.4, 23.0. ESI(+)-MS (m/z): 361 [M + H]^+^. Anal. Calcd for C_21_H_20_N_4_O_2_: C, 69.60; H, 5.01; N, 7.73. Found C, 69.40; H, 4.84; N, 7.51.

### Bioassays

### *In vitro* anti-proliferation assay

*In vitro* cell viability assays were carried out using 96-well microtiter culture plates and 3-(4,5-dimethylthiazol-2-yl)-2,5-diphenyltetrazolium bromide (MTT) staining, according to the standard procedures. S180, HepG_2_, A172, U2OS, A549, Bel-7402/5-Fu, L02, and Cos 7 cells (5 × 10^4^ cells/mL) were obtained either from the Lineberger Cancer Center (UNC-CH) or from ATCC (Rockvile, MD) and were grown in DMEM or RPMI-1640 medium [containing 10% (v/v) fetal calf serum; 60 μg/mL of penicillin, and 100 μg/mL of streptomycin]. Stock solution of DEBIC in DMSO was prepared and diluted with culture medium to desired concentrations. Cultures were propagated at 37°C in a humidified atmosphere (with 5% CO_2_) for 24 hours, and then DEBIC solution was added. After 48 hours of treatment, MTT solution was added (5 mg/mL; 25 μL per well), and cells were incubated for an additional 4 hours. The optical density was measured at 540 nm by a microplate reader, after adding 120 μL of DMSO to dissolve the MTT-formazan product. The mean IC_50_ is the concentration of the derivatives that reduces cell growth by 50% under the experimental conditions and is the average from six independent determinations that were reproducible and statistically significant.

### *In vivo* anti-tumor assay on S180 mice

Male ICR mice (20 ± 2 g), purchased from Animal Center of Capital Medical University, were maintained at 21°C with a natural day/night cycle in a conventional animal colony. S180 ascites tumor cells were used to form solid tumors after subcutaneously injection. For initiation of subcutaneous tumors the cells were obtained as an ascitic form from the tumor-bearing mice, which were serially transplanted once per week. Subcutaneous tumors were implanted by injecting 0.2 mL of 0.9% saline containing 2 × 10^7^ viable tumor cells under the skin on the right armpit. Twenty four hours after implantation, the mice (12 per group) were randomly divided into experimental groups. Doxorubicin is a known intercalator and was selected as the positive control to clarify the validity of the mouse model and the similar action mechanism of the derivatives. The mice of the positive control group were given a daily i.p injection of 8.9 μmol/kg/day of doxorubicin in 0.2 mL of normal saline (NS) for seven consecutive days or 2 μmol/kg/day of doxorubicin in 0.2 mL of normal saline (NS) for nine consecutive days. The mice of the negative control group were given a daily oral administration of 0.2 mL of 0.5% CMC-Na for seven or nine consecutive days. The mice of the treatment groups were given a daily oral administration of 8.9 μmol/kg/day of the derivatives in 0.2 mL of 0.5% CMC-Na for seven consecutive days or given a daily oral 2 μmol/kg/day of the derivative in 0.2 mL of 0.5% CMC-Na for nine consecutive days. The weights of the mice were recorded everyday. Twenty-four hours after the last administration, all mice were weighed, sacrificed by diethyl ether anesthesia and dissected to immediately obtain and weigh the tumor and spleen samples.

### *In vivo* anti-tumor assay on A549 BABL/C mice

A549 cells were collected and resuspended in PBS (pH 7.4), approximately 1 × 10^7^ cells were injected subcutaneously into the hind flank region of the male BALB/C nude mice (25 ± 2 g, purchased from Animal Center of Capital Medical University) and the tumors were allowed to grow. When the tumor perpendicular diameter reached about 5–9 mm, the mice were randomly divided into 3 groups (each 12). Oral 8.9 μmol/kg/day of DEBIC was administered everyday for 9 days. 2 μmol/kg/day of doxorubicin was i.p injected twice a week for 9 days. Each mouse in blank control was given a daily 0.2 mL of 0.5% CMC-Na for nine consecutive days. The weights of the mice were recorded everyday. Twenty-four hours after the last administration, all mice were weighed, sacrificed by diethyl ether anesthesia and dissected to immediately obtain and weigh the tumor and organ samples.

### Intercalation of DEBIC towards CT DNA

### UV spectrum based interaction of DEBIC towards CT DNA

UV spectrum assay can visualize the intercalation of DEBIC towards CT DNA, and this assay was performed. In brief, after recording the UV spectra (Shimadzu 2600 spectrophotometer, 200–350 nm wavelength) of DEBIC (20 μM, in pH 7.4 PBS) in the presence of CT DNA (0, 10, 20, 50 and 70 μM in pH 7.4 PBS) or the UV spectra of CT DNA (100 μM, in pH 7.4 PBS) in the presence of DEBIC (0, 10, 30, 50, 60, 70, 80 and 90 μM, in pH 7.4 PBS) the intercalation of DEBIC towards CT DNA was monitored.

### Fluorescent spectrum based interaction of DEBIC towards CT DNA

Fluorescent spectrum assay can visualize the intercalation of DEBIC towards CT DNA, and this assay was performed. In brief, after recording the fluorescent spectra (Shimadzu RF-5310PC spectrofluorometer, 258 nm of fluorescence excitation wavelength) of DEBIC (10 μM, in pH 7.4 PBS) in the presence CT DNA (0, 10, 30 and 50 μM, in pH 7.4 PBS) or 228 nm of fluorescent spectra of solutions of CT DNA (100 μM, in pH 7.4 PBS) in the presence of DEBIC (0, 20, 40 and 100 μM, in pH 7.4 PBS) the intercalation of DEBIC towards CT DNA was monitored.

### Relative viscosity based interaction of DEBIC towards CT DNA

The intercalation of DEBIC towards CT DNA was mirrored with the relative viscosity of CT DNA, and the data were recorded on Ubbeholde viscometer immersed in a thermostated water bath maintained at 25°C. In the assay to a solution of CT DNA in PBS (13 mL, 80 μM) the solutions of DEBIC in PBS were added for keeping the ratio of [DEBIC]:[CT DNA] in the range of 0–0.8 to form the samples, and the flow time of the samples were measured after a thermal equilibrium time of 5 min. The flow time of each sample was calculated. The relative viscosity of CT DNA in the presence and absence of DEBIC were calculated from the equation, *η* = (*t-t*_0_)/*t*_0_, wherein *t*_0_ and *t* were the observed flow time in the absence and presence of DEBIC. Data are presented as (*η*/*η*_0_)^1/3^ versus binding ratio, wherein *η* is the viscosity of CT DNA in the presence of DEBIC and *η*_0_ is the viscosity of CT DNA alone.

### CD spectrum based interaction of DEBIC towards CT DNA

In CD experiments a solution system (pH 7.4) consisting of a solution of CT DNA in PBS buffer (100 µM) and a solution of DEBIC in PBS buffer (0, 20, 40 and 100 µM) were incubated at 37°C for 3 h, and the spectra were tested according to a standard procedure.

### Arterial thrombus weight assay of DEBIC on rat model

Male Sprague-Dawley rats (weight, 200 to 250 g) were randomly divided into groups (each 12). Thirty min after orally giving 0.5% CMC-Na (3 mL/kg) or suspension of aspirin in 0.5% CMC-Na (dose, 16.7 and 167 µmol/kg) or suspension of DEBIC in 0.5% CMC-Na (dose, 0.36 µmol/kg) the rats intraperitoneally received 20% ethyl carbamate sodium anesthesia (1.4 g/kg) to separate the left jugular vein and the right carotid artery. Polyethylene tubes were used to make shunts between the jugular vein and carotid artery. Into the middle polyethylene tube a weighed 6-cm non absorbable surgical thread of silk was put, and a solution of heparin sodium in NS (71.4 IU/mL) was injected as anticoagulant to fill the tube. The ends of the tube were inserted into the left jugular vein and the right carotid artery, and via the tube the blood flowed from the right carotid artery to the left jugular vein for 15 min. The surgical thread was taken out to weigh the thrombus weight, and to sample the blood, the brain, the heart, the lung, the liver, the kidney and the spleen.

### Homogenates based ESI(+)-FT-ICR-MS analysis

To examine the targeting action the thrombus, blood, brain, heart, kidney, liver and spleen of 0.36 μmol/kg oral DEBIC treated rats and the thrombus of 0.5% CMC-Na treated rats were homogenized, the homogenates were extracted with methanol and the extracts were given ESI(+)-FT-ICR-MS analysis. In the preparation of blood extract the blood of DEBIC treated rats received ultrasonication (30 min) and centrifugation (4000 r/min, 15 min). The supernatant was extracted with ethyl acetate (0.5 mL × 10) to extract DEBIC. The ethyl acetate was blow with nitrogen to dry, and the residue was dissolved in chromatographic pure methanol (200 μL methanol per g of blood) for FT-ICR-MS analysis. In the preparation of the extract of thrombus of 0.36 μmol/kg oral DEBIC or 0.5% CMC-Na the thrombus received ultrasonication (30 min) and centrifugation (4000 r/min, 15 min). The supernatant was extracted with ethyl acetate (0.5 mL × 10) to extract DEBIC. The ethyl acetate was blow with nitrogen to dry, and the residue was dissolved in chromatographic pure methanol (200 μL methanol per g of blood) for FT-ICR-MS analysis. In the preparation of the extracts of the organs of 0.36 μmol/kg oral DEBIC the same procedure for preparing thrombus extract was used.

### Arterial thrombus weight assay of DEBIC on mouse model

Male ICR mice (weight, 20 to 25 g) were randomly divided into three groups (12 each). The mice were orally given 0.5% CMC-Na (10 mL/kg) or a suspension of aspirin in 0.5% CMC-Na (240 µmol/kg of dose) or a suspension of DEBIC in 0.5% CMC-Na (0.36 µmol/kg of dose). Thirty min after administration the mice were anesthetized with ethyl carbamate (15 g/100 mL, intraperitoneally), then 1 cm of segment abdominal aorta was dissected. To trigger thrombosis immediately the artery of the mice was wrapped in gauze strips (0.5 cm in width and 3 cm in length) saturated with 25% aqueous ferric chloride for 15 min to damage the vessel, 0.5 cm of artery segment with thrombi formation was excised, and the weight of the thrombus was determined.

### AFM image and the effect of DEBIC on platelets

Rat blood and 3.8% aqueous sodium citrate (9/1, v/v) was centrifuged at 1000 rpm for 10 min to collect the platelet-rich plasma (PRP), then PRP was centrifuged for another 10 min at 3000 rpm to collect the precipitates. To the precipitates 1.5 mL NS was added, the mixture was centrifuged (3000 rpm/min, 10 min) to get resting platelets (10^5^/mL). The resting platelets were incubated with DEBIC (10^−2^, 10^−4^ and 10^−6^ mg/L) or NS at 37°C for 30 min. The resting platelets were activated with AA (0.4 mM) at 37°C for 5 min. AA activated platelets were incubated with DEBIC (10^−2^, 10^−4^ and 10^−6^ mg/L) or NS at 37°C for 30 min. The platelet samples were individually dropped onto a mica sheet, fixed with glutaraldehyde (3%) for 10 min, carefully washed with ultrapure water, and dried in air. With the contact mode AFM images of platelet samples and DEBIC (10^−2^, 10^−4^ and 10^−6^ mg/L) were recorded on a Nanoscope 3D AFM (Veeco Metrology, Santa Barbara, CA, USA) in ambient conditions.

### UV based P-selectin expression assay

Sample diluents for P-selectin expression assay were from rat ELISA kit (Rat P-selectin ELISA Kit; Wuhan Huamei Biotech Co., Ltd., Wuhan, Hubei Province, People’s Republic of China). A solution of P-selectin in sample diluents was prepared (300 ng/mL). Into an eppendorf tube 300 μL of this solution was added. The control tube only contains 300 μL of sample diluents. The sample tube contains 300 μL solution of P-selectin in sample diluents (300 ng/mL) and 10 μL solution of DEBIC in sample diluents (2, 4, 6, 8 and 10 μM). All tubes were incubated at room temperature for 12 h then received UV test on a Shimadzu 2550 spectrophotometer.

### ELISA based P-selectin expression assay

Blood of the rats treated with 0.36 µmol/kg DEBIC or 167 µmol/kg aspirin or 0.5% CMC-Na was collected into a tube containing 3.8% sodium citrate at a ratio of 9/1 and at 1000 rpm centrifuged for 30 min to immediately collect the serum. This serum received 10-fold-dilution with the diluents from the ELISA kit (Rat P-selectin ELISA Kit; Wuhan Huamei Biotech Co., Ltd., Wuhan, Hubei Province, People’s Republic of China) to get 980 μL of serum sample. The serum sample was at 37°C incubated for 3 min to prepare the blank or test serum. To the control well and the test well of a 96- well plate coated with the enzyme, 100 μL blank or test serum were added, respectively. The wells were at 37°C incubated for 120 min. After removing the solvent, 100 μL of the biotin labeling antibody (from the kit) was added to each well and then at 37°C incubated for 60 min. The solution of each well was discarded, 200 μL of washing solution (from the kit) was added to wash the plate for three times. After adding 100 μL/well of horseradish peroxidase labeling avidin (from the kit), at 37°C the plate was incubated for 60 min, and then washed five times. For coloration, to each well 90 μL of the substrate solution (from the kit) was added and then at 37°C incubated in dark for 30 min. To stop the reaction 50 μL/well of the stop solution (from the kit) was added. The OD value of the well was measured at 450 nm within 15 min of the addition of the stop solution. Consequently, the P-selectin level was calculated according to the standard samples (from the kit).

## CONCLUSIONS

Arterial thrombosis has been known as one of the major complications of cancer and can seriously worsen the prognosis of the patients. In the onset of cancer and the complication of arterial thrombosis both P-selectin and DNA are likely involved. These findings hypothesize that an agent could simultaneously inhibit P-selectin and intercalates DNA. By structural analysis of P-selectin inhibitors and DNA intercalators of bisindoles 12 derivatives of bisindolediacetic acids were designed. The scores of them docking the active sites of P-selectin and d(CGATCG)_2_ led to the synthesis, and the *in vivo* anti-tumor evaluation of 9 derivatives. The docking score and the anti-tumor activity *in vivo* consistently benefit DEBIC. A series of evaluations ensured that DEBIC was capable of slowing tumor growth and inhibiting arterial thrombosis in a targeting manner. Thus DEBIC shows the cross talk between tumor growth, arterial thrombosis, P-selectin expression, DNA intercalation and platelet activation, and thereby explores the advantage of P-selectin inhibition and DNA intercalation in chemical therapy of cancer patients to prevent the complication of arterial thrombosis.

## SUPPLEMENTARY MATERIALS FIGURES AND TABLE


