# Supplementary material for: Discovery of DEBIC to correlate P-selectin inhibition and DNA intercalation in cancer therapy and complicated thrombosis
Source: Oncotarget. 2017 Dec 8;9(63):32119–33. doi: 10.18632/oncotarget.23151 (PMC6114953; doi:10.18632/oncotarget.23151)
Supplement: Supplementary file 1 [file oncotarget-09-32119-s001.pdf]

Discovery of DEBIC to correlate P-selectin inhibition and DNA intercalation in cancer therapy and complicated thrombosis

SUPPLEMENTARY MATERIALS

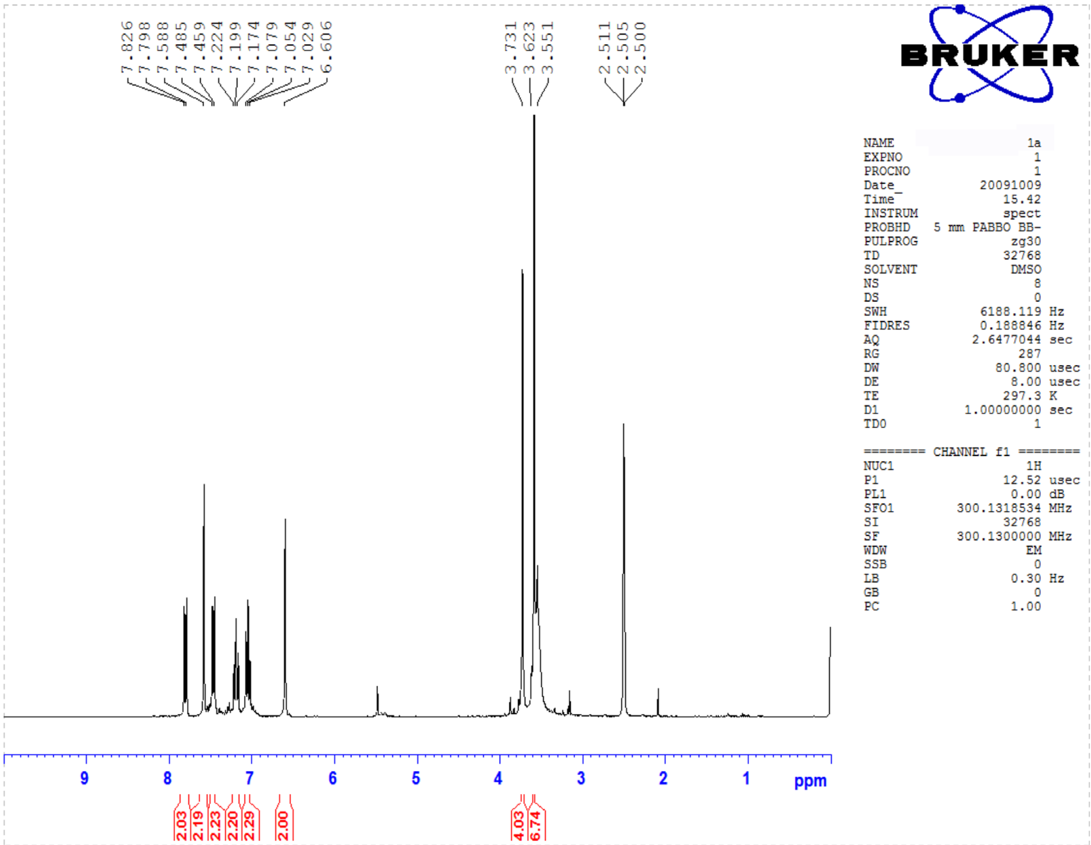

Supplementary Figure 1: ^{1}H-NMR spectrum of 1a (300 MHz, DMSO-*d6*).

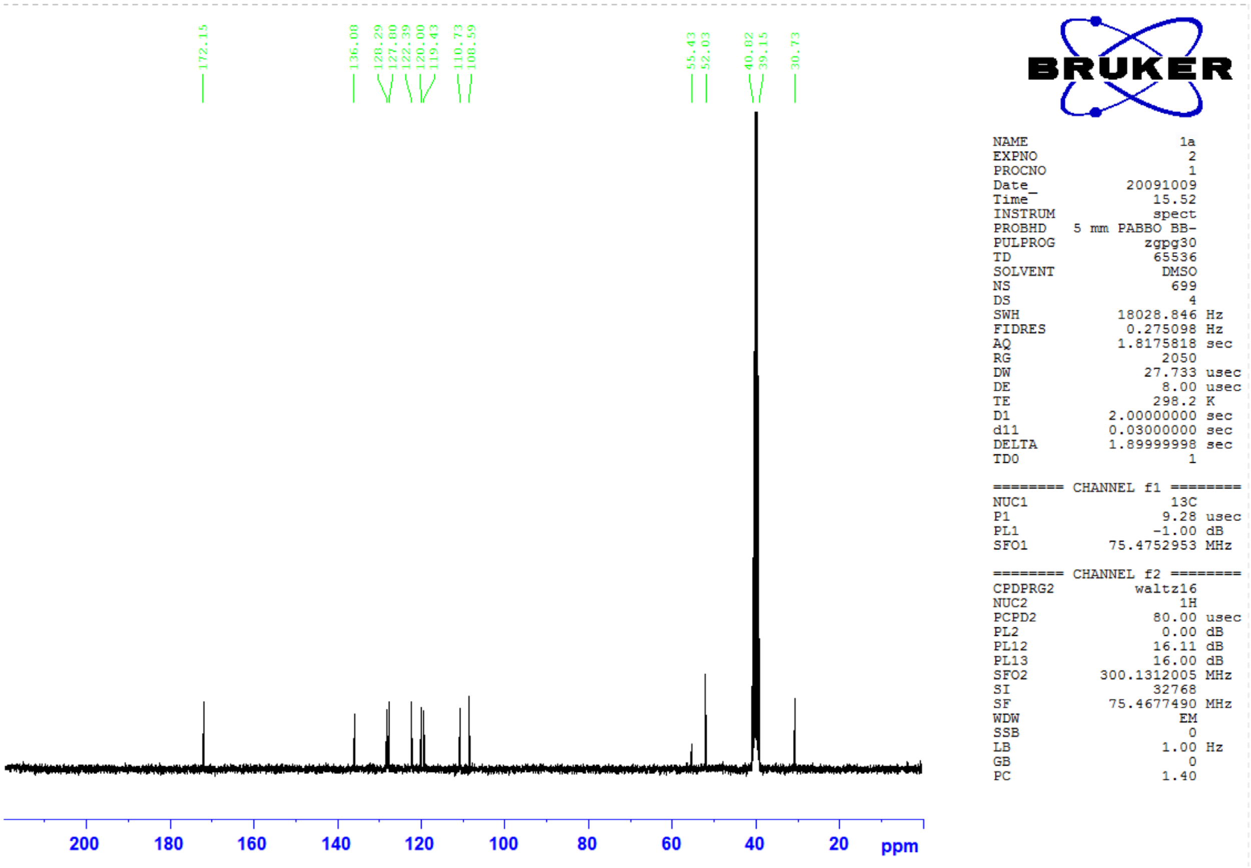

Supplementary Figure 2: ^{13}C-NMR spectrum of 1a (75 MHz, DMSO-*d6*).

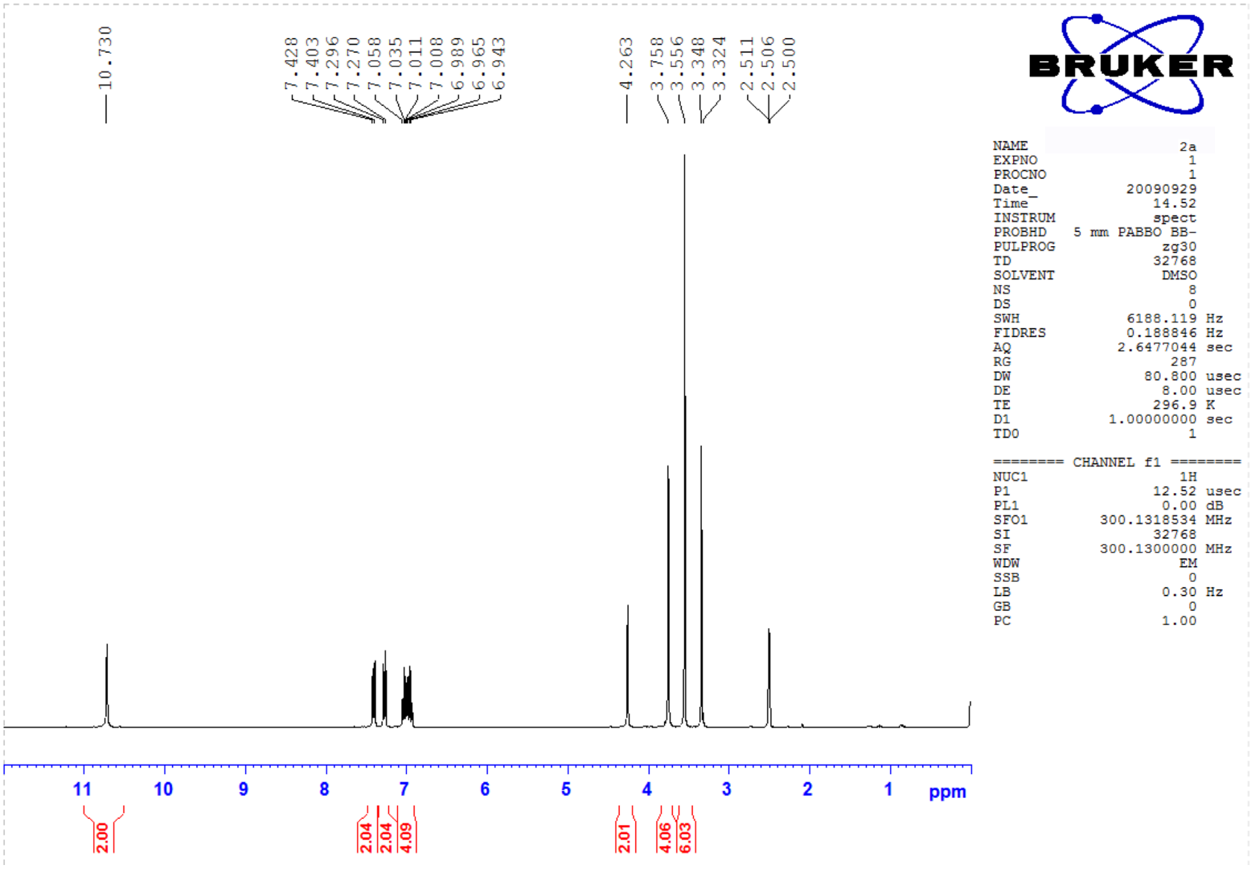

Supplementary Figure 3: 1H-NMR spectrum of 2a (300 MHz, DMSO-*d6*).

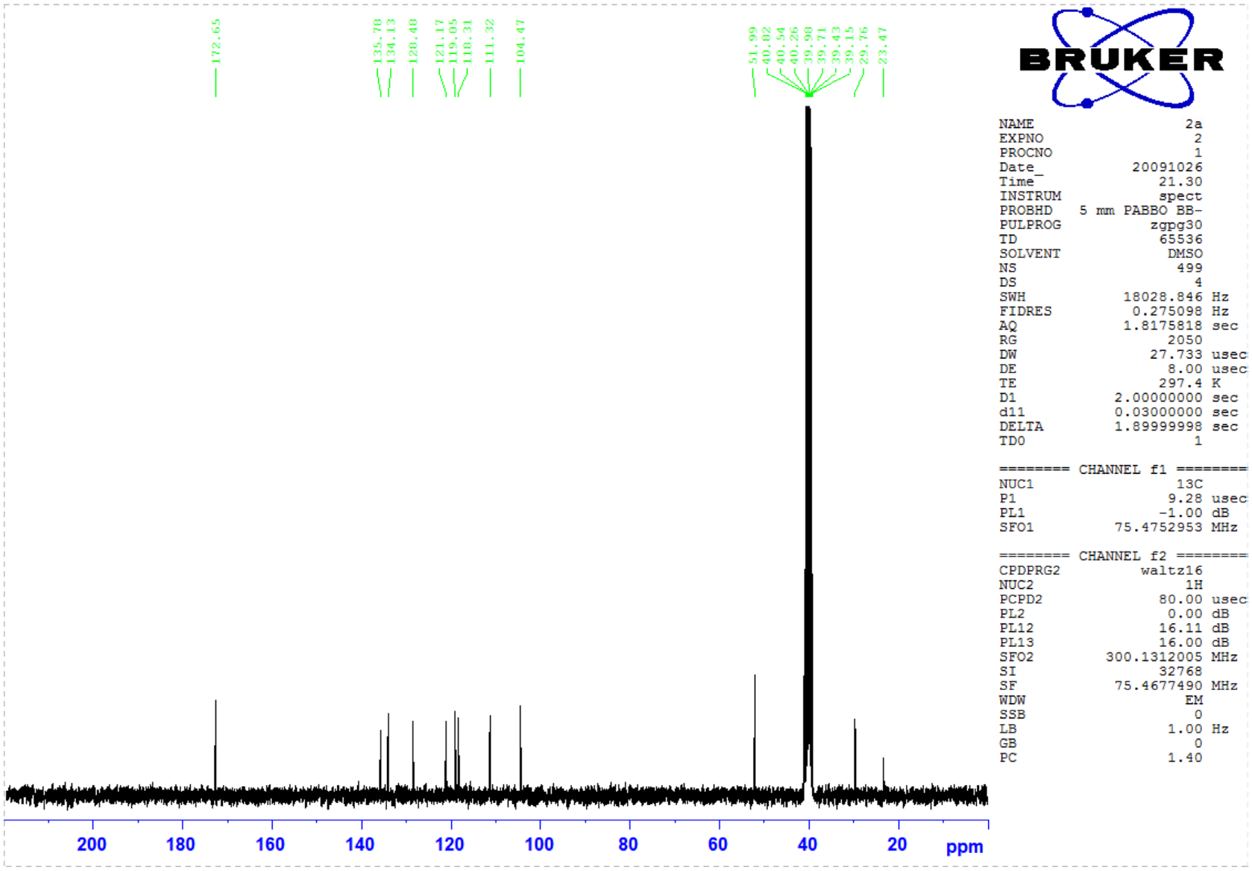

Supplementary Figure 4: ^{13}C-NMR spectrum of 2a (75 MHz, DMSO-*d6*).

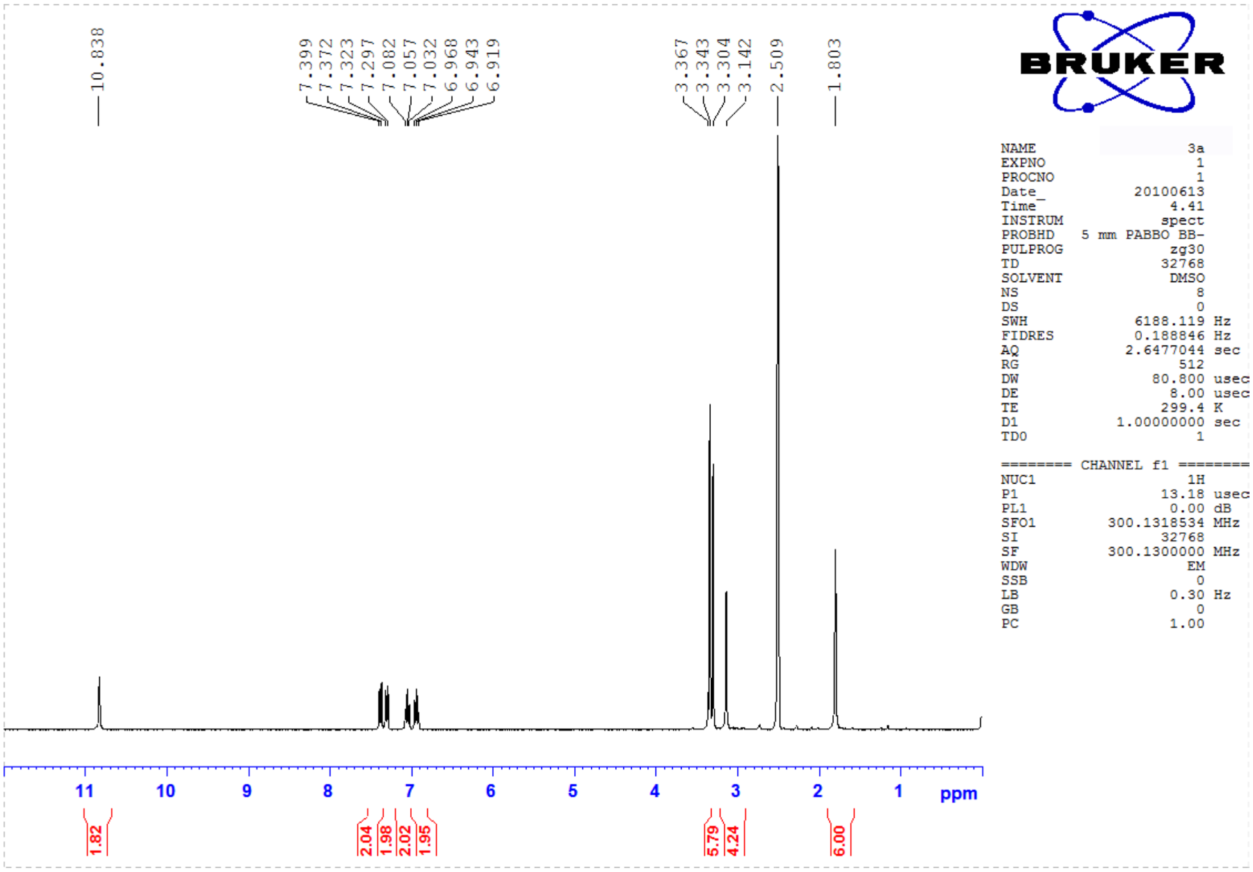

Supplementary Figure 5: ¹H-NMR spectrum of 3a (300 MHz, DMSO-*d6*).

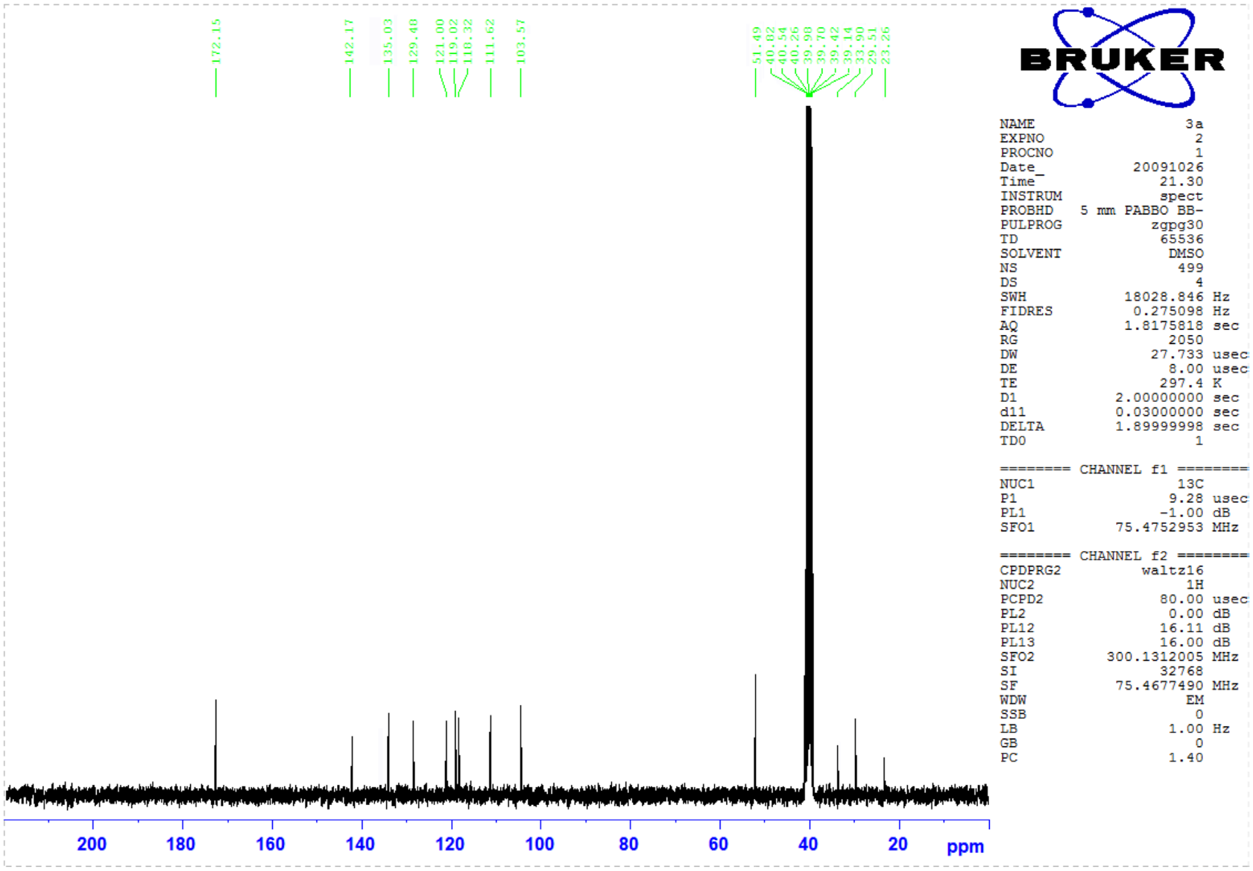

Supplementary Figure 6: ^{13}C-NMR spectrum of 3a (75 MHz, DMSO-*d6*).

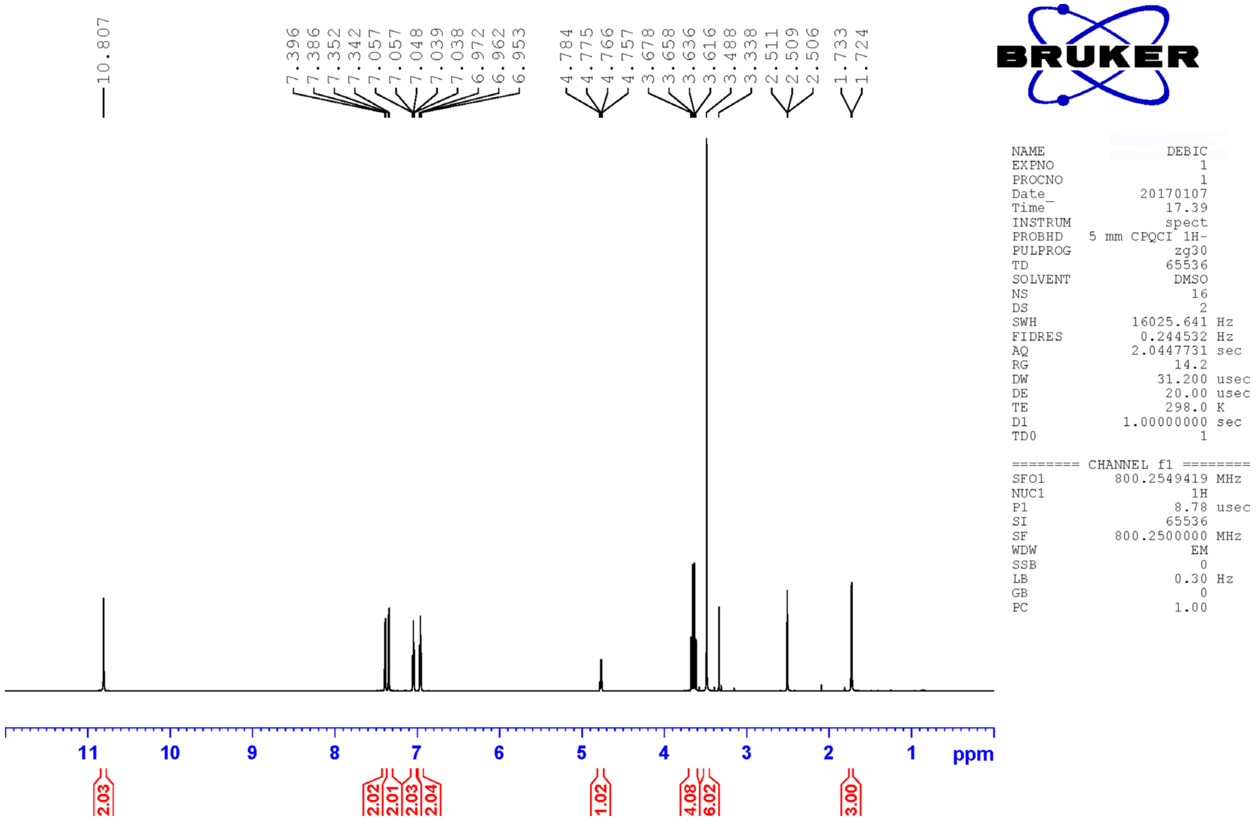

Supplementary Figure 7: ^{1}H-NMR spectrum of DEBIC (800 MHz, DMSO-*d6*).

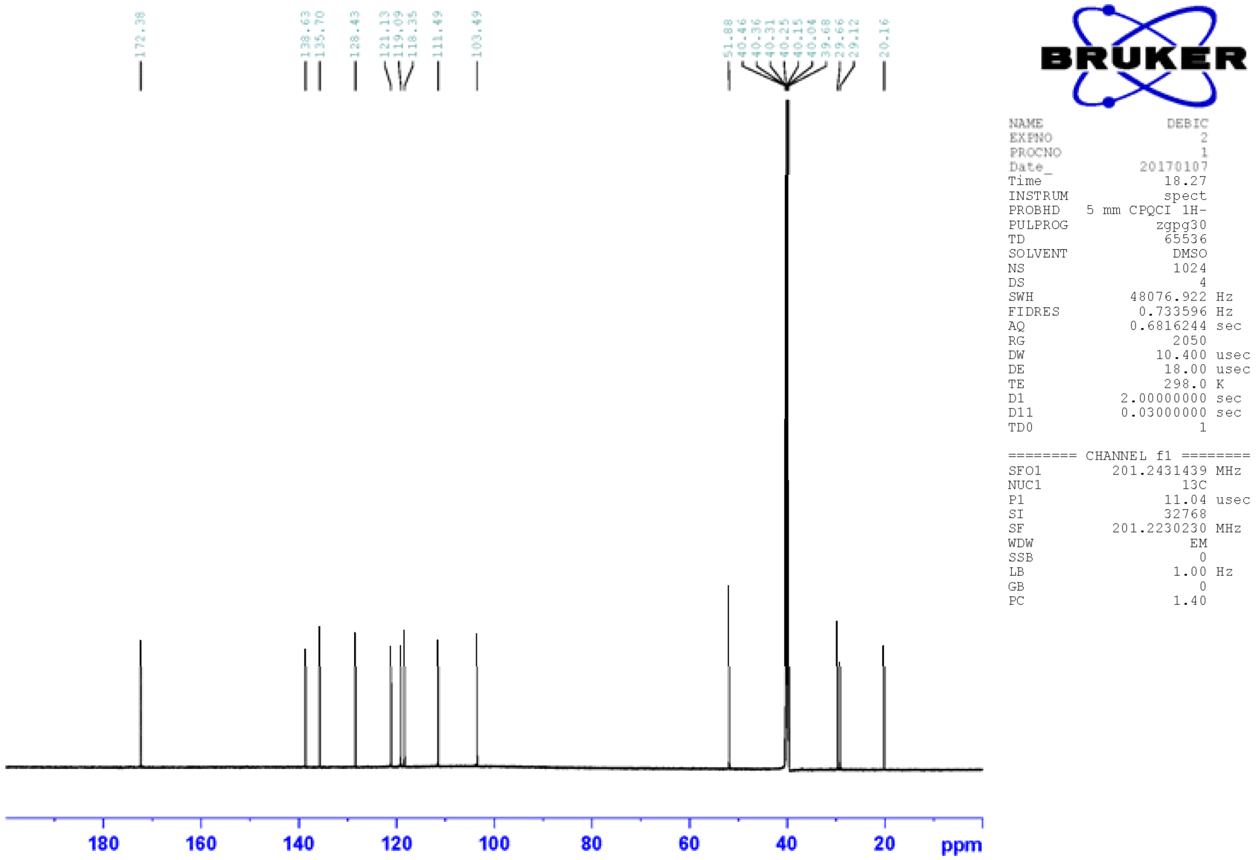

Supplementary Figure 8: ^{13}C-NMR spectrum of DEBIC (200 MHz, DMSO-*d6*).

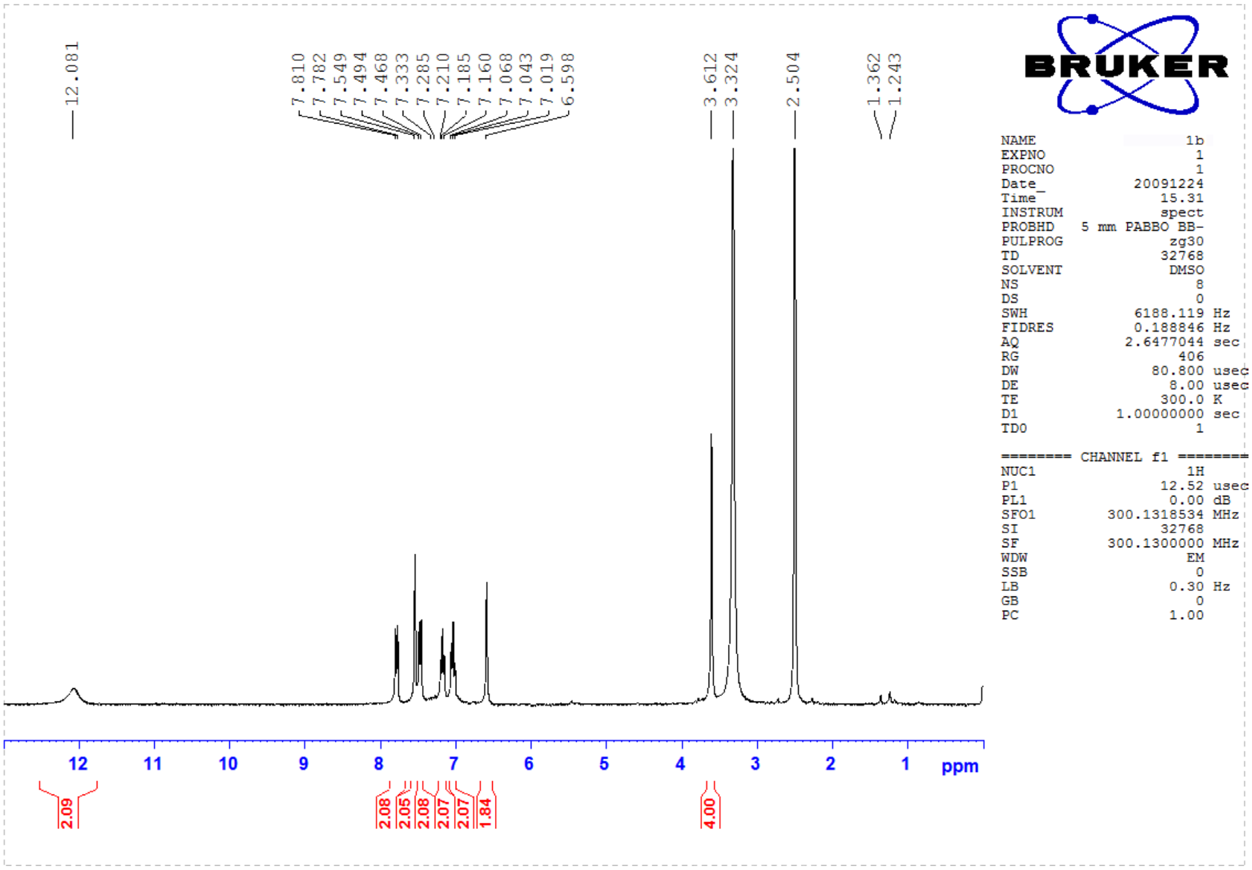

Supplementary Figure 9: ^{1}H-NMR spectrum of 1b (300 MHz, DMSO-*d6*).

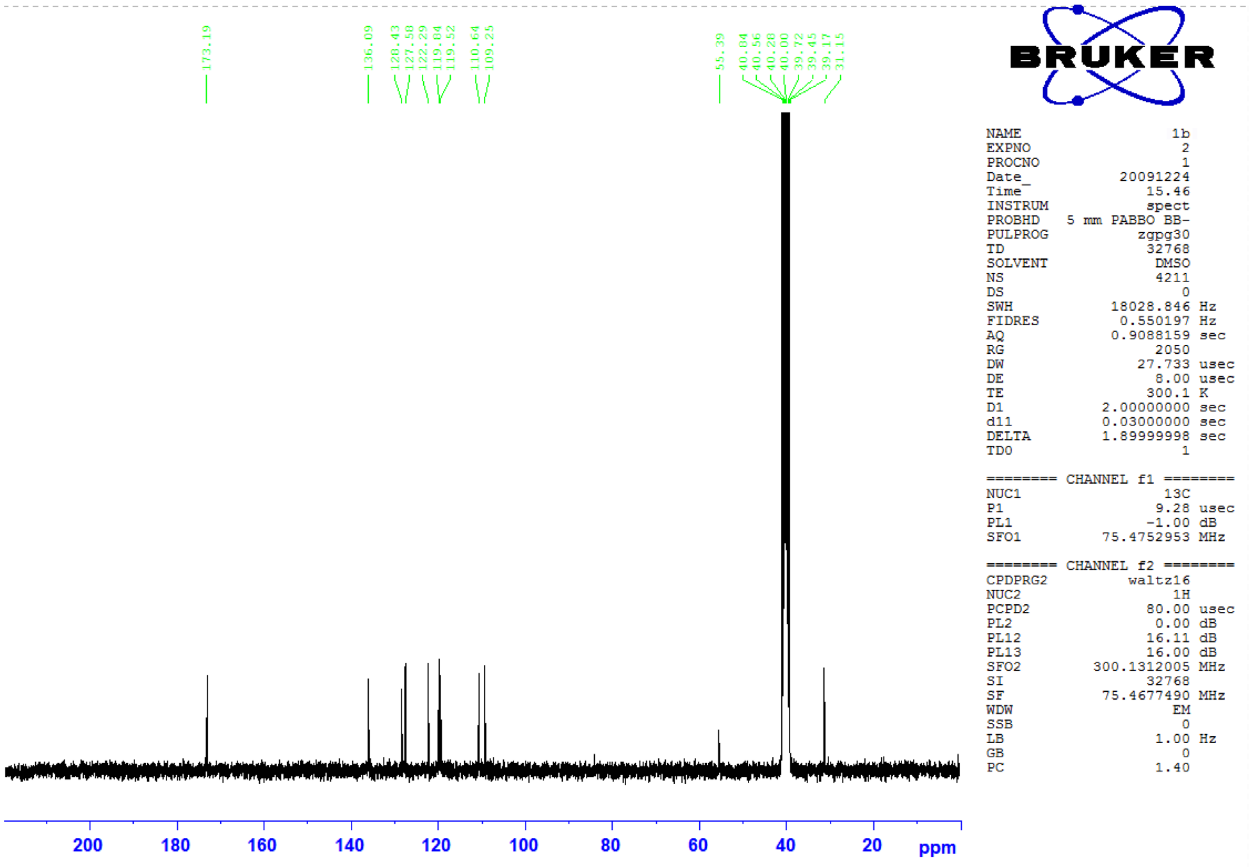

Supplementary Figure 10: ^{13}C-NMR spectrum of 1b (75 MHz, DMSO-*d6*).

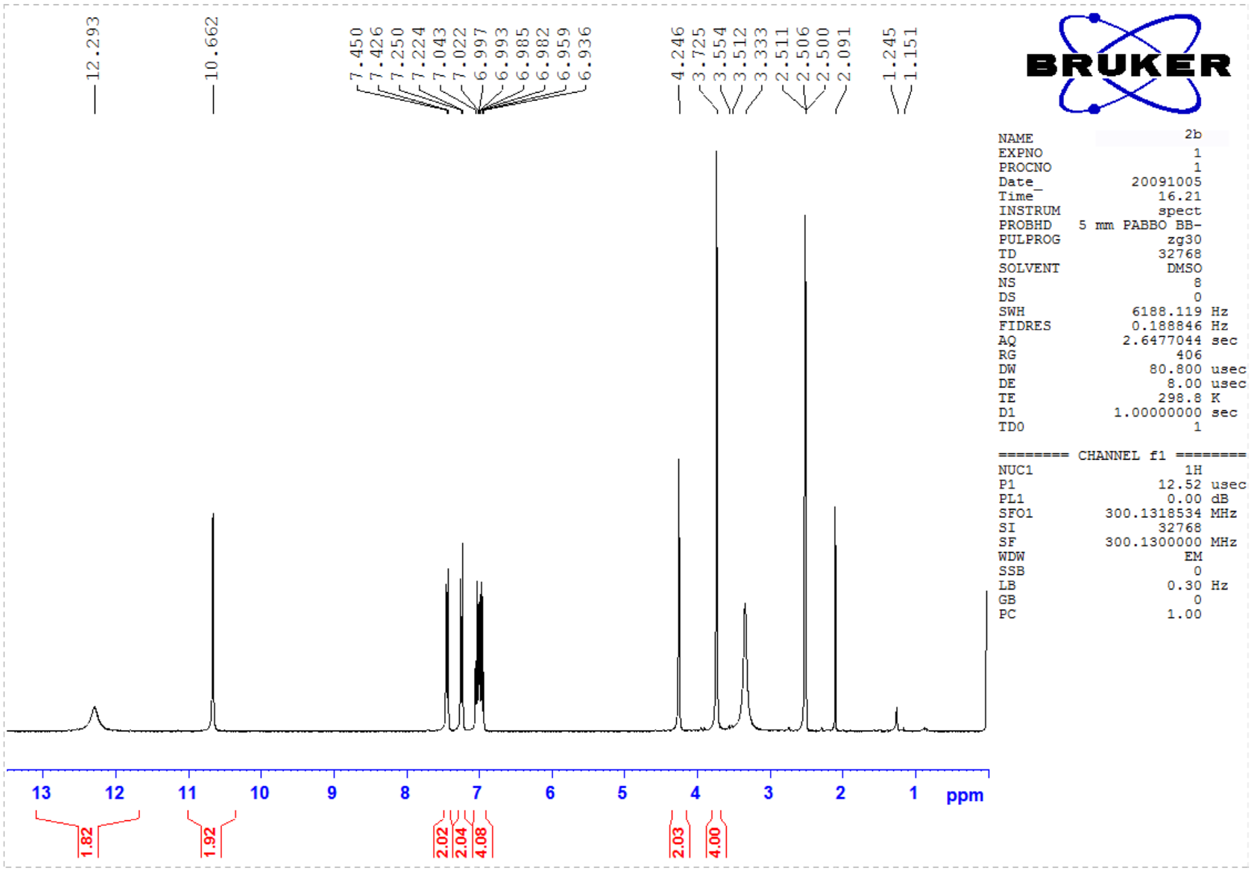

Supplementary Figure 11: ^{1}H-NMR spectrum of 2b (300 MHz, DMSO-*d6*).

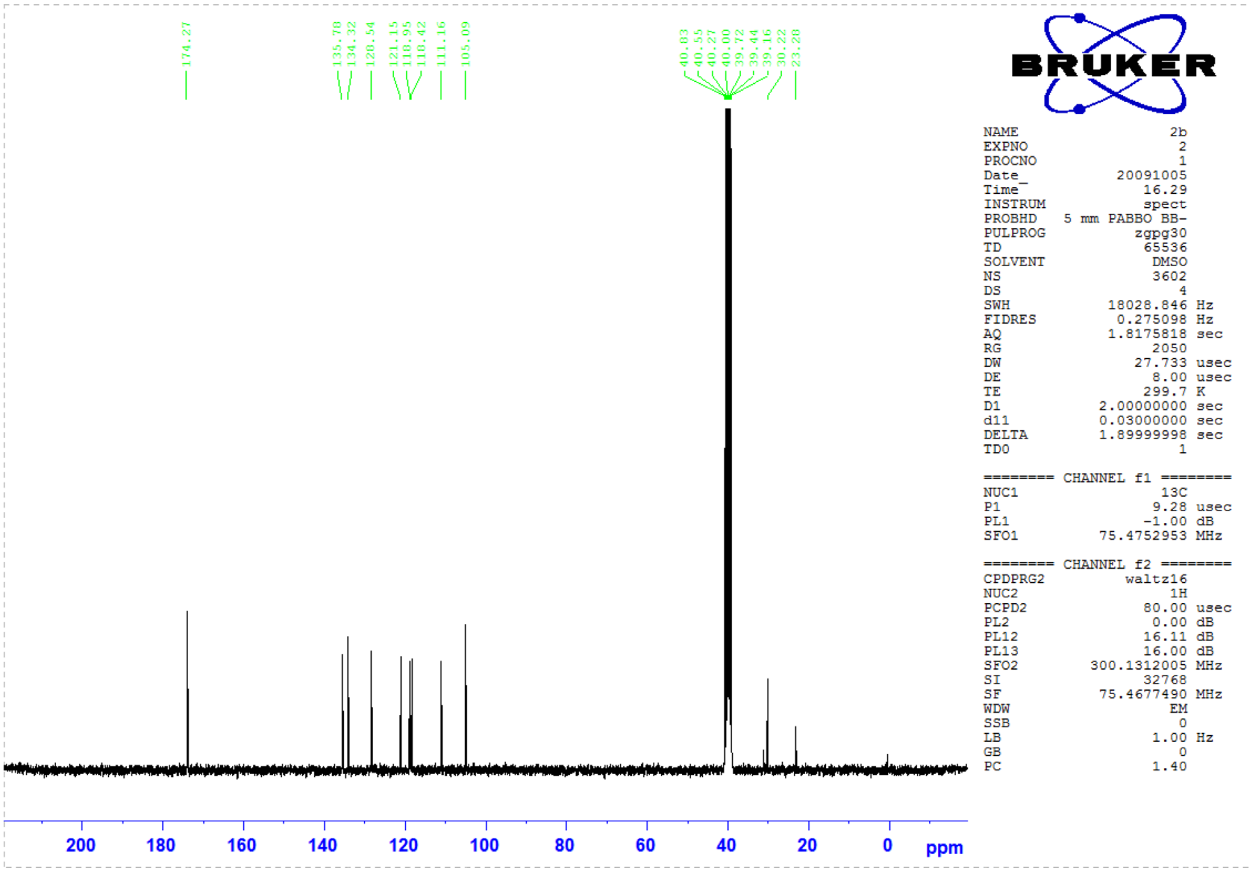

Supplementary Figure 12: ^{13}C-NMR spectrum of 2b (75 MHz, DMSO-*d6*).

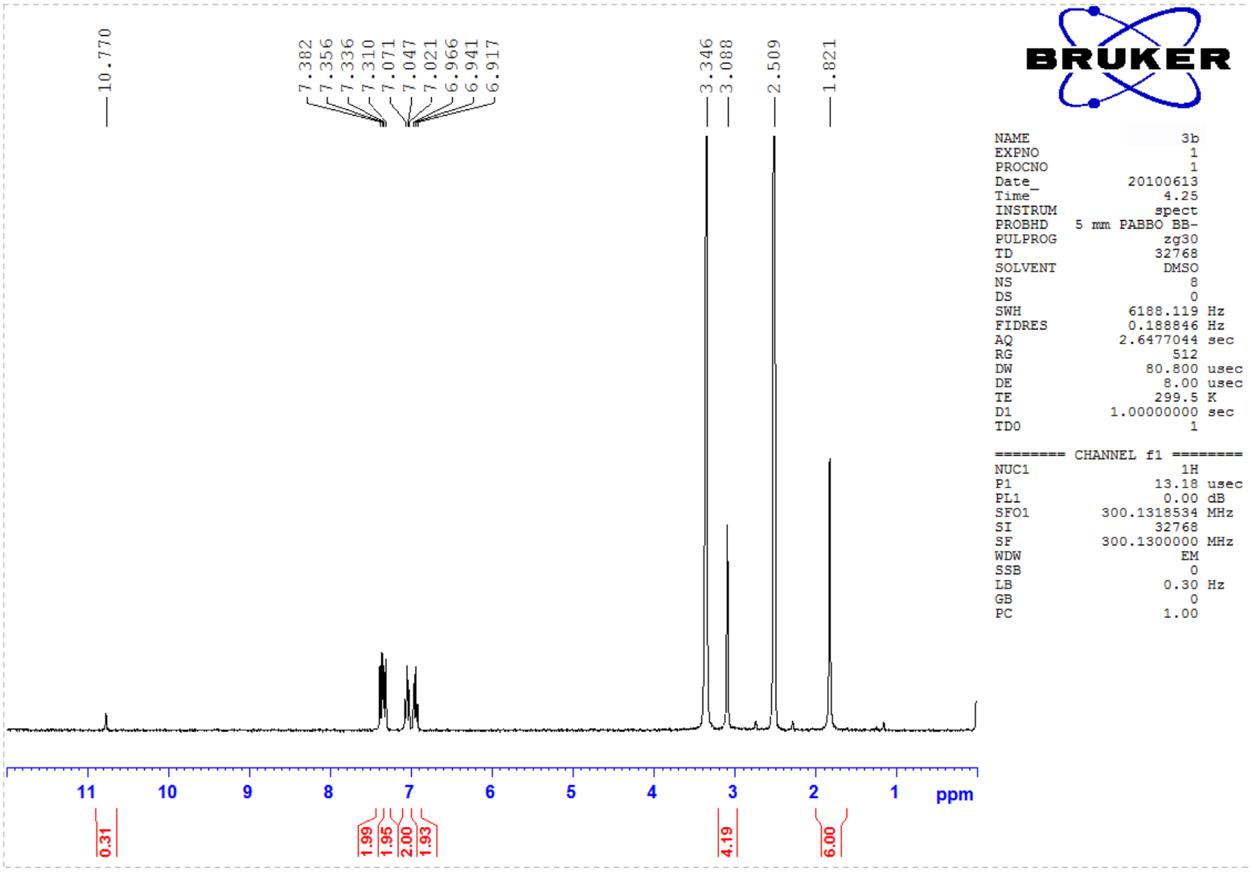

Supplementary Figure 13: ^{1}H-NMR spectrum of 3b (300 MHz, DMSO-*d6*).

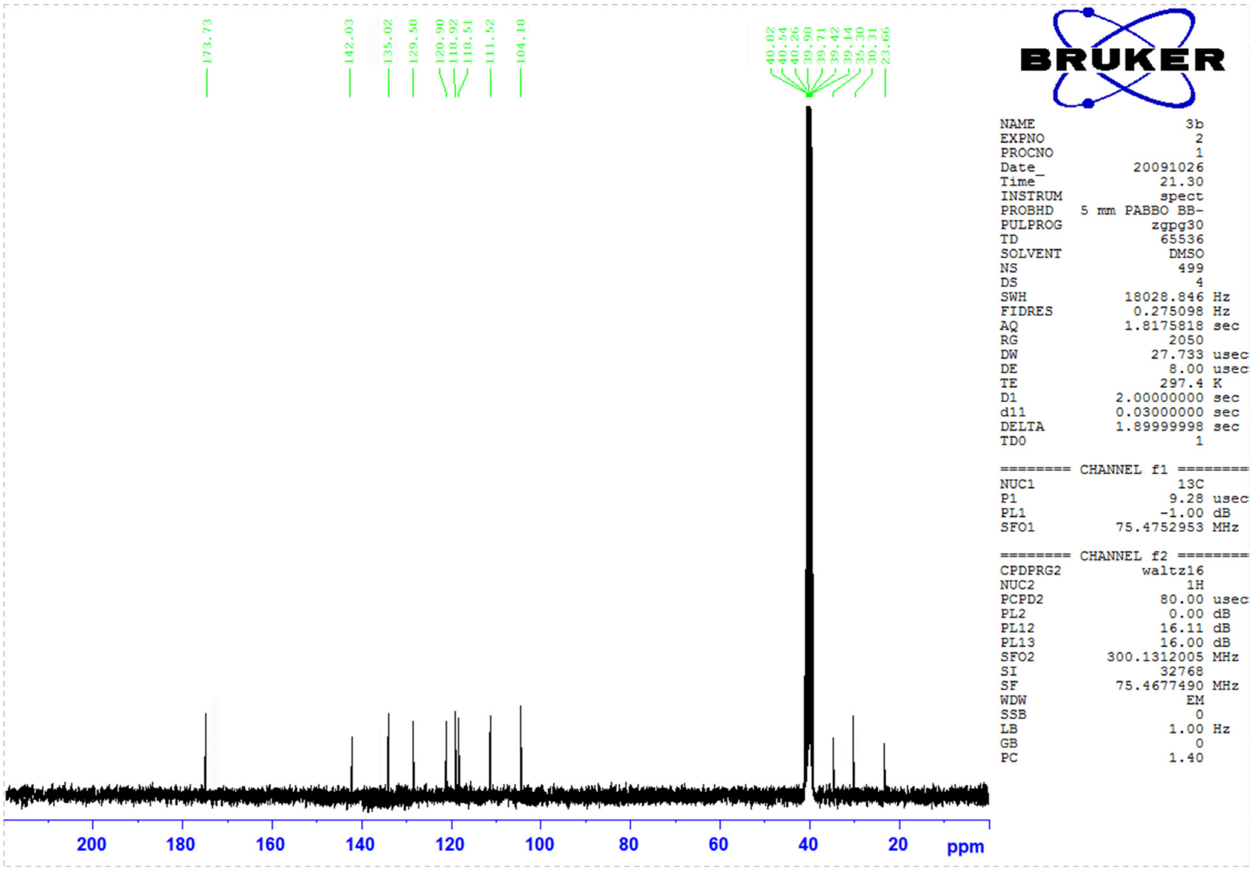

Supplementary Figure 14: ^{13}C-NMR spectrum of 3b (75 MHz, DMSO-*d6*).

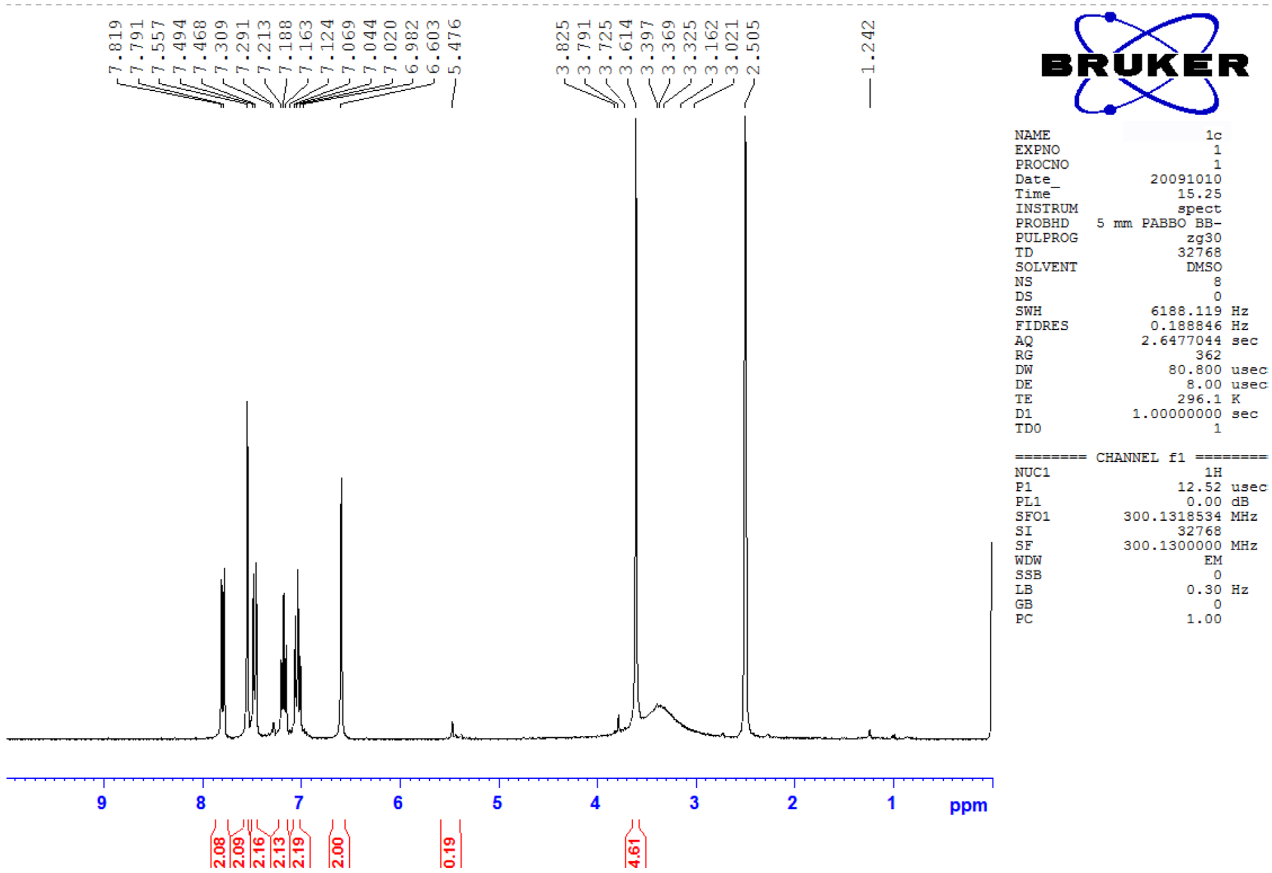

Supplementary Figure 15: ^{1}H-NMR spectrum of 1c (300 MHz, DMSO-*d6*).

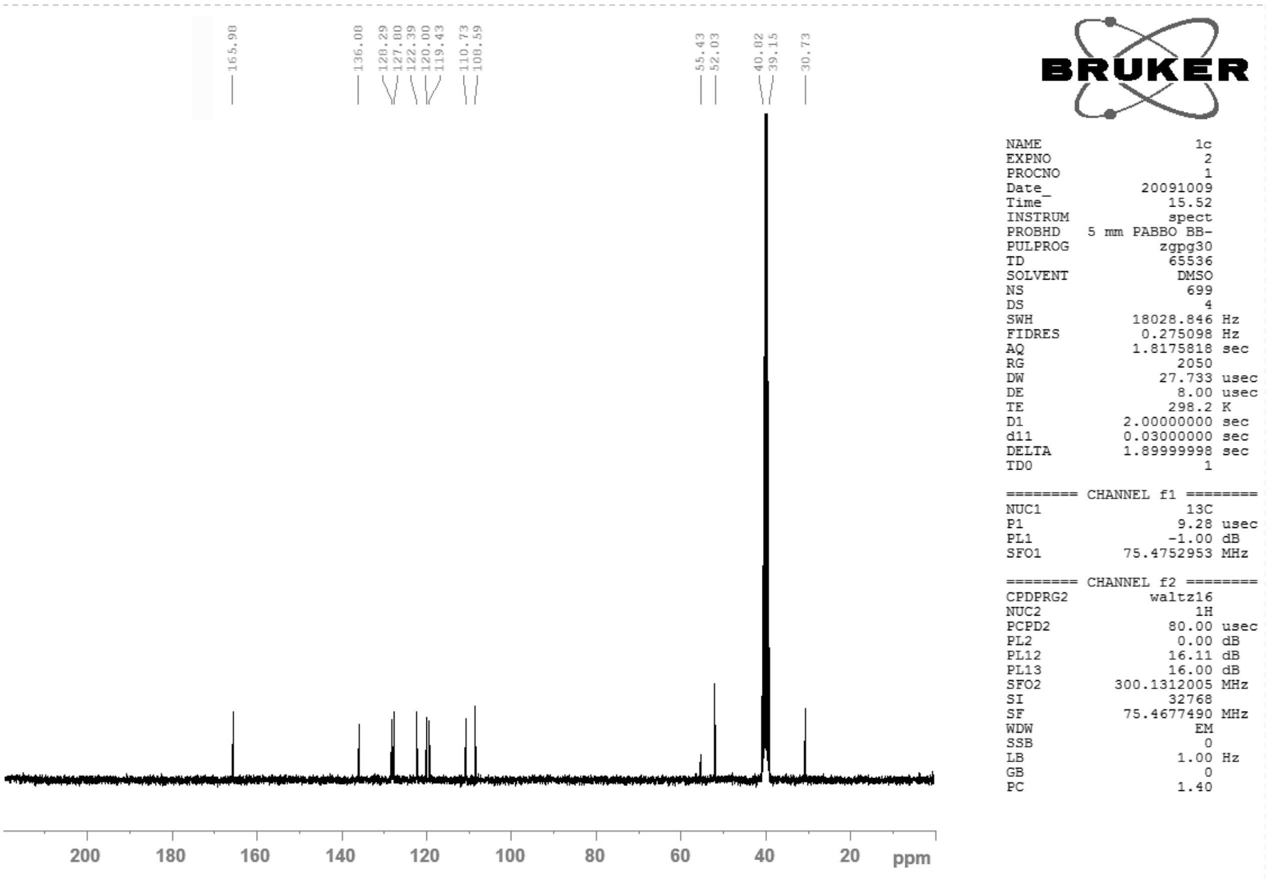

Supplementary Figure 16: ^{13}C-NMR spectrum of 1c (75 MHz, DMSO-*d6*).

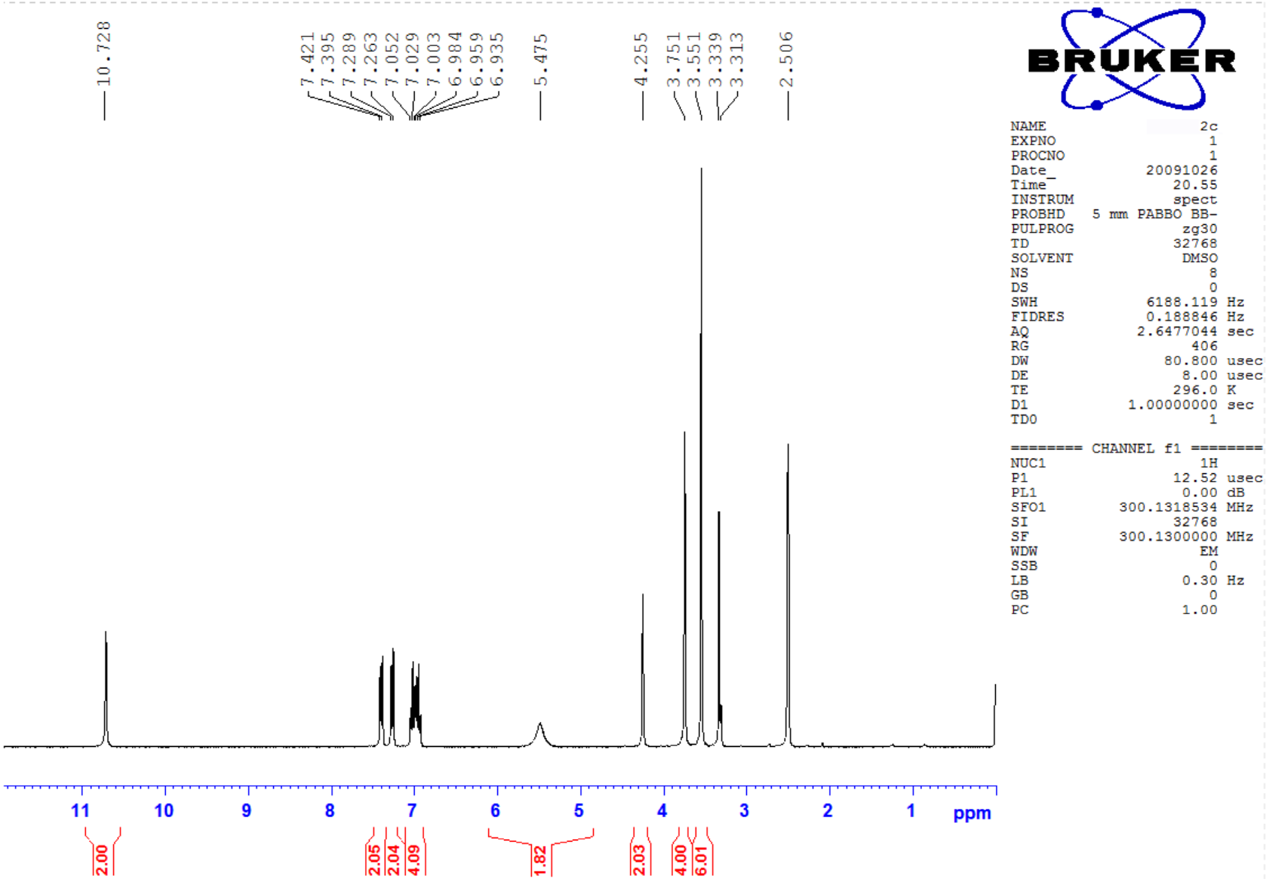

Supplementary Figure 17: ^{1}H-NMR spectrum of 2c (300 MHz, DMSO-*d6*).

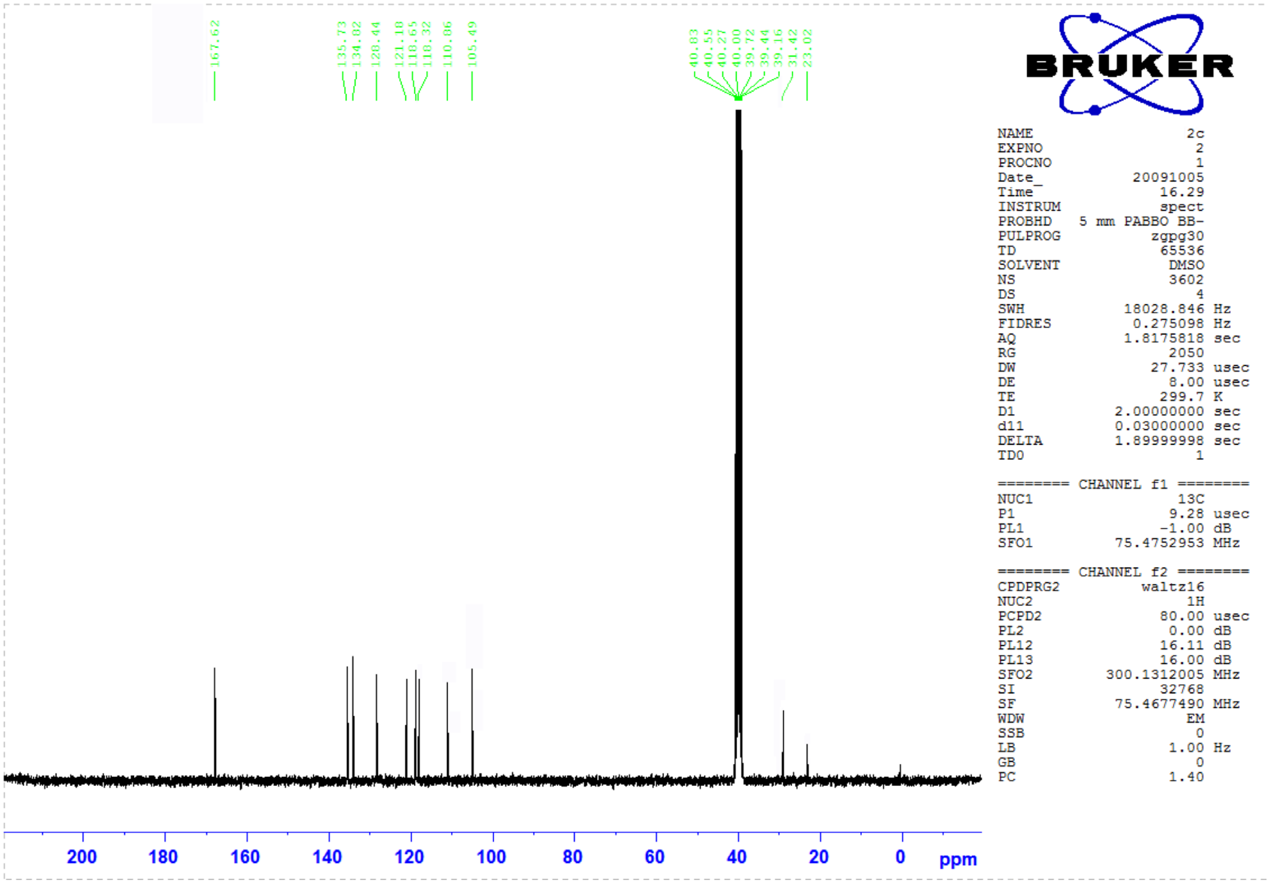

Supplementary Figure 18: ^{13}C-NMR spectrum of 2c (75 MHz, DMSO-*d6*).

Supplementary Table 1: SMILE Document

Compounds	SMILE	S180 tumor weight (8.9 µmol/kg/day, Mean ± SD g)	S180 tumor weight (1.7 µmol/kg/day, Mean ± SD g)	S180 tumor weight (0.36 µmol/kg/day, Mean ± SD g)	A549 tumor weight (8.9 µmol/kg/day, Mean ± SD g)	Mouse artcrial thrombus weight (0.36 µmol/kg/day, Mean ± SD mg)	Rat artcrial thrombus weight (0.36 µmol/kg/day, Mean ± SD mg)
1a	O=C(OC([H])([H])[H])C([H])([H])C1=C([H])N(C2=C1C([H])=C([H])C([H])=C2[H])C([H])([H])N4/C3=C(\[H])C([H])=C([H])C([H])=C3/C(=C4/[H])C([H])([H])C(=O)OC([H])([H])[H]	1.53 ± 0.15					
2a	O=C(OC([H])([H])[H])C([H])([H])C1=C(N([H])C2=C1C([H])=C([H])C([H])=C2[H])C([H])([H])C=4N([H])C3=C([H])C([H])=C([H])C([H])=C3C=4C([H])([H])C(=O)OC([H])([H])[H]	1.26 ± 0.31					
3a	O=C(OC([H])([H])[H])C([H])([H])C=2C=1/C(=C(/[H])C([H])=C([H])C=1[H])N([H])C=2C(C=3N([H])C4=C(C=3C([H])([H])C(=O)OC([H])([H])[H])C([H])=C([H])C([H])=C4[H])(C([H])([H])[H])C([H])([H])[H]	0.69 ± 0.21					
DEBIC	O=C(OC([H])([H])[H])C([H])([H])C=2C=1/C(=C(/[H])C([H])=C([H])C=1[H])N([H])C=2[C@@]([H])(C=3N([H])C4=C(C=3C([H])([H])C(=O)OC([H])([H])[H])C([H])=C([H])C([H])=C4[H])C([H])([H])[H]	0.36 ± 0.21	0.59 ± 0.12	0.90 ± 0.32	0.68 ± 0.09	1.19 ± 0.34	19.06 ± 4.38
1b	O=C(O[H])C([H])([H])C1=C([H])N(C2=C1C([H])=C([H])C([H])=C2[H])C([H])([H])N4/C3=C(\[H])C([H])=C([H])C([H])=C3/C(=C4/[H])C([H])([H])C(=O)O[H]	0.92 ± 0.25					
2b	O=C(O[H])C([H])([H])C1=C(N([H])C2=C1C([H])=C([H])C([H])=C2[H])C([H])([H])C=4N([H])C3=C([H])C([H])=C([H])C([H])=C3C=4C([H])([H])C(=O)O[H]	0.85 ± 0.26					
3b	O=C(O[H])C([H])([H])C1=C(N([H])C2=C1C([H])=C([H])C([H])=C2[H])C(C=4N([H])C3=C([H])C([H])=C([H])C([H])=C3C=4C([H])([H])C(=O)O[H])(C([H])([H])[H])C([H])([H])[H]	0.79 ± 0.18					
1c	O=C(N([H])[H])C([H])([H])C1=C([H])N(C2=C1C([H])=C([H])C([H])=C2[H])C([H])([H])N4/C3=C(\[H])C([H])=C([H])C([H])=C3/C(=C4/[H])C([H])([H])C(=O)N([H])[H]	1.22 ± 0.29					
2c	O=C(N([H])[H])C([H])([H])C1=C(N([H])C2=C1C([H])=C([H])C([H])=C2[H])C([H])([H])C=4N([H])C3=C([H])C([H])=C([H])C([H])=C3C=4C([H])([H])C(=O)N([H])[H]	1.50 ± 0.28					
